# Non-genetic remodeling drives leukemia propagation and reveals actionable vulnerabilities in acute myeloid leukemia

**DOI:** 10.1016/j.xcrm.2026.103017

**Published:** 2026-08-31

**Authors:** Clement Larrue, Paolo Angelino, Sarah Mouche, Alexa S. Green, Emeline Boet, Ismael Boussaid, Claudia Perez-Carretero, Rudy Birsen, Maxime Sajot, Thomas A. Mckee, Ambrine Sahal, Nicolas Chapuis, François Vergez, Christian Recher, Véronique Mansat-De Mas, Olivier Kosmider, Pierre Sujobert, Frederic Catez, Jean-Jacques Diaz, Jean-Emmanuel Sarry, Petros Tsantoulis, Jerome Tamburini

**Affiliations:** 1 Translational Research Centre in Onco-hematology, Faculty of Medicine, University of Geneva, and Swiss Cancer Center Leman, Geneva, Switzerland; 2 Université de Toulouse, Centre de Recherches en Cancérologie de Toulouse, Inserm U1037, CNRS U5077, équipe labellisée Ligue Nationale Contre le Cancer 2023, LabEx Toucan, Toulouse, France; 3 Translational Data Science, SIB Swiss Institute of Bioinformatics, Lausanne, Switzerland; 4 Université Paris-Cité, Institut Cochin, CNRS U8104, Inserm U1016, Paris, France; 5 Pathology Department, Geneva University Hospital, Geneva, Switzerland; 6 Université de Toulouse, Centre Hospitalier Universitaire de Toulouse, Institut Universitaire du Cancer de Toulouse Oncopole, Service d'Hématologie, 31100 Toulouse, France; 7 Hospices Civils de Lyon, Hôpital Lyon Sud, Service d'Hématologie Biologique, Lyon, France; 8 Ribosome, Translation and Cancer Team, LabEx DEVweCAN, Institut Convergence Plascan, LYriCAN+, Centre de Recherche en Cancérologie de Lyon, INSERM U1052, CNRS UMR5286, Centre Léon Bérard, Université de Lyon, Université Claude Bernard Lyon 1, 69008 Lyon, France

**Keywords:** acute myeloid leukemia, leukemic stem cells, patient-derived xenografts, serial xenotransplantation, translational regulation, ribosome profiling, DNA methylation, drug screening

## Abstract

Acute myeloid leukemia (AML) persistence and relapse are sustained by leukemia-propagating cells, yet the molecular programs supporting their expansion during disease evolution remain incompletely understood. Using serial patient-derived xenotransplantation, we establish a longitudinal model in which leukemia-initiating capacity progressively increases. Integrated single-cell transcriptomics and multi-omics profiling reveal a predominantly non-genetic trajectory that follows a conserved pattern across models and is associated with coordinated changes across epigenetic, transcriptional, and proteomic layers. Ribosome profiling and rRNA 2′-O-methylation analyses further support a stage-specific increase in translational activity with ribosome remodeling in advanced xenografts. A pharmacological screen of 3,247 compounds uncovers a limited set of vulnerabilities that consistently emerge during disease progression, including CRBN-dependent degradation of GSPT1 (CC-885) and IAP antagonism (AZD5582). *In vivo* validation shows that both agents markedly reduce leukemic burden, impair leukemia propagation, and enhance cytarabine activity in patient-derived xenograft (PDX) models. Together, these findings show that leukemic propagation is driven by a non-genetic remodeling program, providing a framework to prioritize and test stage-specific therapeutic strategies in AML.

## Highlights

• Serial xenotransplantation progressively enriches leukemia-propagating capacity in AML
• Single-cell and multi-omics profiling define a non-genetic evolutionary trajectory
• Translational activation and ribosome remodeling mark advanced xenograft stages
• CC-885 and AZD5582 suppress leukemia propagation and enhance cytarabine *in vivo*

## Graphical abstract

**Figure:**
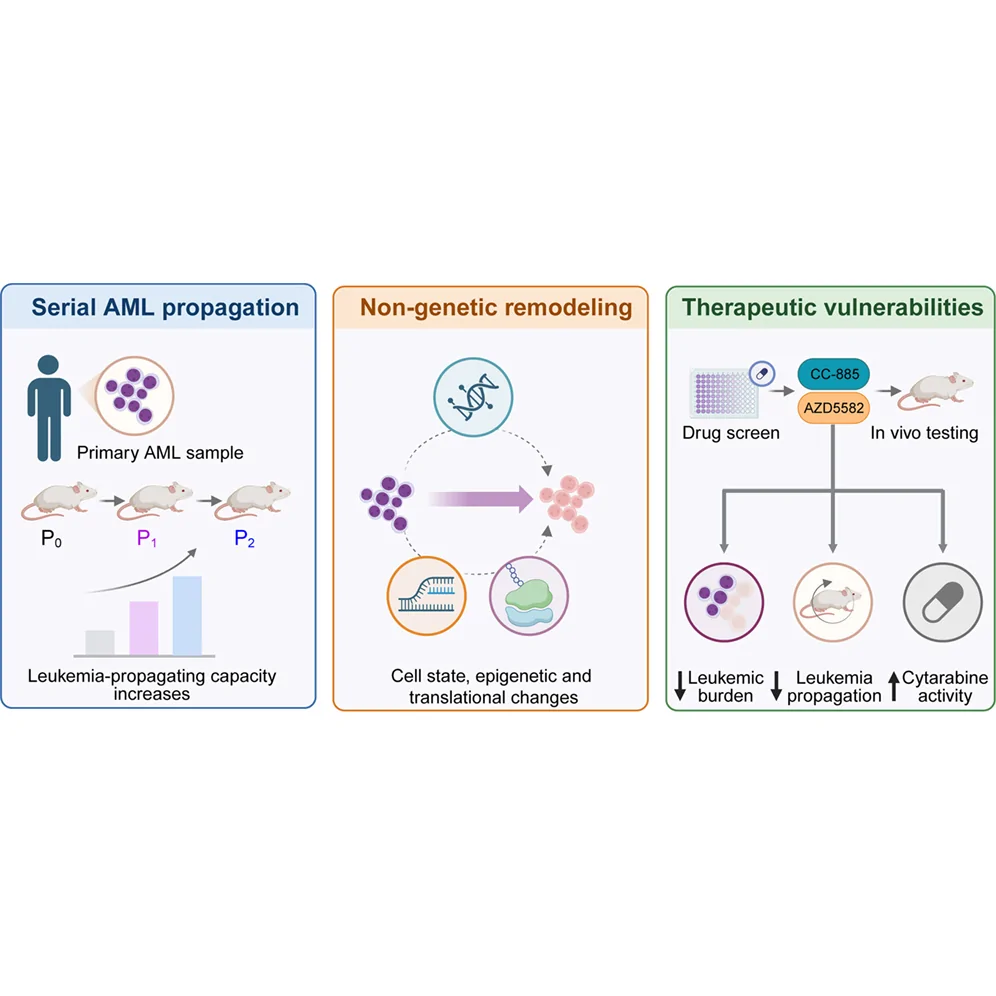

## Teaser

Larrue et al. show that serial propagation of AML in xenografts increases leukemia-propagating activity and is accompanied by molecular and drug-response changes. Integrated profiling links these changes to translational and ribosome programs and identifies therapeutic candidates that limit leukemia propagation *in vivo*.

## Introduction

Acute myeloid leukemia (AML) is characterized by frequent relapse and limited long-term survival, particularly in older adults and patients with refractory disease.^1^^,^^2^ Disease persistence and recurrence are sustained by leukemic cells capable of reconstituting leukemia after therapy.^3^^,^^4^ These propagation-competent cells are operationally defined as leukemic stem cells (LSCs) by their ability to initiate and maintain disease in transplantation assays.^3^^,^^5^^,^^6^ In immunodeficient mice, this property is measured using patient-derived xenograft (PDX) models, where only leukemia-initiating cells sustain long-term engraftment.^7^^,^^8^ In these functional settings, LSCs have been associated with transcriptional and epigenetic programs that promote quiescence, metabolic adaptability, and resistance to cytotoxic stress.^9^^,^^10^^,^^11^ Recent single-cell studies have further shown that AML is organized along a continuum of transcriptional states reflecting normal hematopoietic differentiation hierarchies.^12^

While this framework robustly defines LSC activity, it does not explain the molecular programs that sustain disease propagation during evolution. Surface markers only partially capture leukemia-initiating potential, and functional stemness does not consistently align with immunophenotype.^8^^,^^13^ Although relapse often reflects expansion of leukemic populations with self-renewal capacity,^14^^,^^15^ it remains unclear whether progressive enrichment of propagation-competent states is driven primarily by genetic diversification or by coordinated non-genetic remodeling.

We hypothesized that serial *in vivo* transplantation would progressively enrich propagation-competent leukemic states and reveal the molecular adaptations underlying their selection within the murine xenograft microenvironment. To test this, we established a longitudinal PDX model in NOD/LtSz-SCID/IL-2Rγchain null (NSG) mice and performed integrated profiling of matched primary and xenografted AML samples using genomics, DNA methylation, transcriptomics, proteomics, ribosome profiling, and large-scale pharmacological screening. This design enabled direct comparison of genetic and non-genetic changes across successive passages.

Serial transplantation led to stepwise enrichment of leukemia-initiating capacity without substantial genetic diversification. Single-cell transcriptomics revealed reproducible reorganization of leukemic cellular states, with progressive accumulation of proliferative and biosynthetic programs. Integrated analyses across molecular layers identified coordinated epigenetic, transcriptional, and post-transcriptional remodeling, with reinforcement of translational capacity emerging as a defining feature of advanced xenografts. Screening of 3,247 compounds uncovered evolution-enriched therapeutic vulnerabilities, including CRBN-dependent degradation of GSPT1 and IAP antagonism.^16^^,^^17^ *In vivo* validation showed that targeting these dependencies reduced leukemic burden, impaired reconstitution capacity, and enhanced cytarabine (Ara-C) activity.

Together, these findings provide a longitudinal view of leukemic adaptation, in which non-genetic remodeling reinforces propagation and exposes therapeutically exploitable vulnerabilities in AML.

## Results

### Serial xenotransplantation enriches leukemia-initiating cell capacities in immunodeficient mice

**Figure 1:**
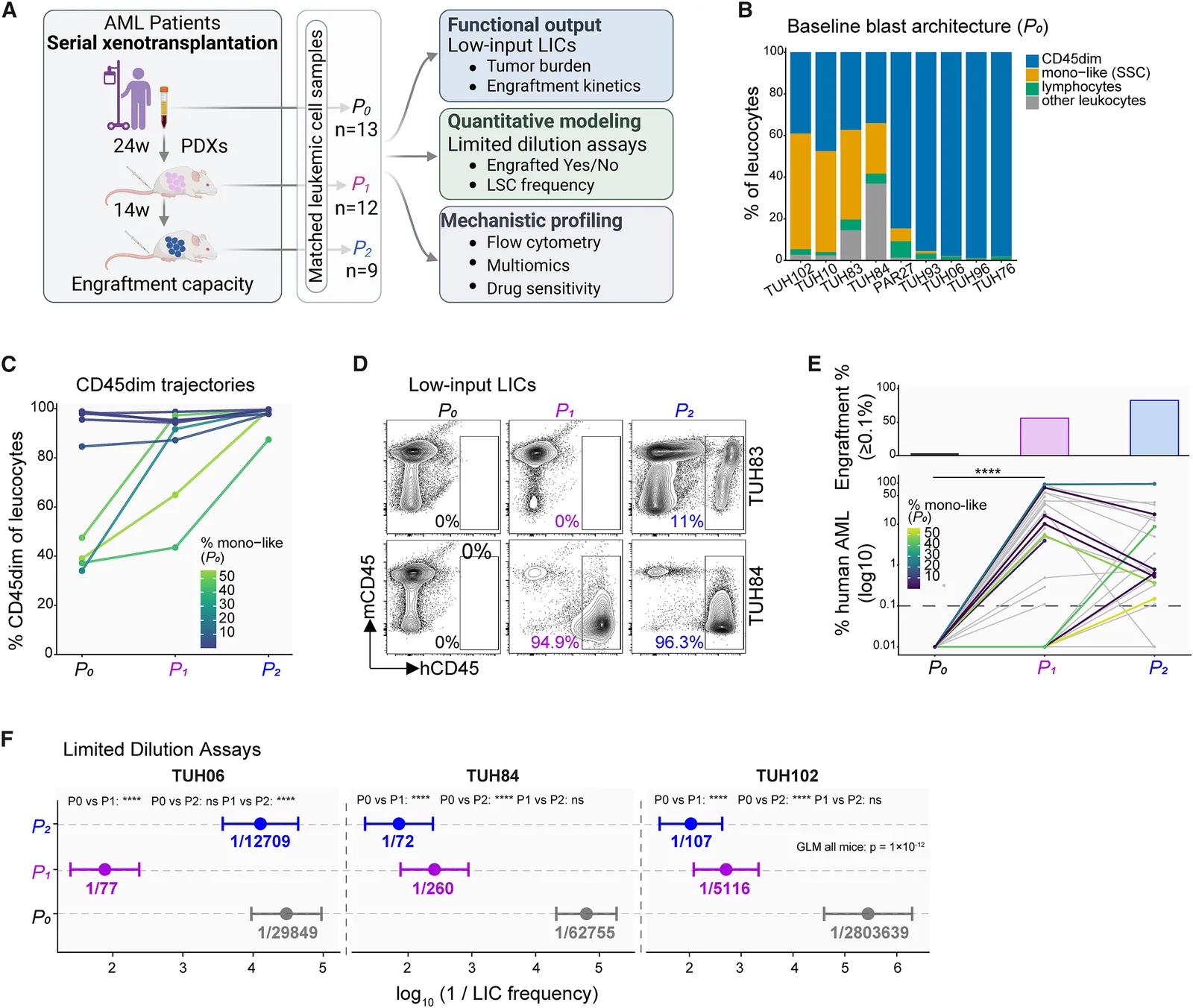
Serial xenotransplantation enriches leukemia-initiating cell capacities in immunodeficient mice (A) Experimental design. Cryopreserved AML samples obtained at diagnosis (*P*_*0*_; *n* = 13) were transplanted into NSG mice to generate primary xenografts (*P*_*1*_; *n* = 12; ∼24 weeks), followed by secondary transplantation to establish *P*_*2*_ xenografts (*n* = 9; ∼14 weeks). Matched *P*_*0*_-*P*_*1*_-*P*_*2*_ samples were subjected to functional assays (low-input leukemia-initiating cell [LIC] assays, limiting dilution assays), flow cytometry, and downstream molecular profiling. (B) Baseline leukocyte composition at diagnosis. Stacked bar plots show the proportion of CD45_dim_ blasts, SSC-defined mono-like cells, lymphocytes, and other leukocytes within each primary AML sample. (C) Longitudinal changes in leukemic architecture across passages. The fraction of CD45_dim_ blasts (percentage of human CD45^+^ cells) is shown for each model at *P*_*0*_, *P*_*1*_, and *P*_*2*_. Lines connect matched samples across passages; color intensity reflects the initial mono-like fraction at *P*_*0*_. *n* = 9 matched trios. (D) Representative flow cytometry plots (murine CD45 versus human CD45) from low-input LIC assays in TUH83 and TUH84 at *P*_*0*_, *P*_*1*_, and *P*_*2*_, illustrating progressive engraftment capacity. (E) Top: engraftment frequency in low-input LIC assays (threshold ≥0.1% human CD45^+^). Bottom: leukemic burden (% human AML cells; log_10_ scale) in recipient mice across passages. Each dot represents one mouse; lines connect matched models (*n* = 9 matched trios). (F) Limiting dilution analysis (LDA) in TUH06, TUH84, and TUH102 across *P*_*0*_, *P*_*1*_, and *P*_*2*_ (*n* = 4 mice per cell dose). Estimated LIC/LSC frequencies (log_10_ scale, 1/frequency) with confidence intervals are shown. GLM, generalized linear models. Statistical comparisons between passages are indicated. Statistical significance: ∗∗∗∗*p* < 0.0001.

Serial xenotransplantation is commonly used to identify leukemic clones capable of sustaining long-term disease propagation.^5^^,^^6^ We used PDX models in NOD/SCID/IL-2R-γchain null (NSG) mice to follow leukemia-initiating activity across successive *in vivo* passages and to generate matched samples for downstream analyses (Figure 1A). Viable cryopreserved blood or bone marrow samples from thirteen newly diagnosed AML patients (*P*_*0*_) were transplanted into NSG mice (Table 1). To preserve the cellular diversity present at diagnosis, we transplanted blast-enriched mononuclear fractions obtained by density gradient separation, without isolating specific immunophenotypic subsets, even though LSC activity is often enriched within the CD34^+^CD38^−^ fraction.^4^^,^^8^^,^^18^ Primary xenografts (*P*_*1*_) were established in 12 cases after 24 weeks,^19^ and 9 were further propagated during 14 weeks to secondary recipients (*P*_*2*_), generating 9 matched trios for longitudinal study (Figure 1A). Samples were selected from our internal PDX collection, based on prior engraftment capacity, which likely explains the high overall engraftment rate from primary samples (Table 1).

**Table 1:** Clinical and molecular characteristics of patients with AML

Patient	G	Age	W	B	LSC	FAB	Karyotype	NGS	ELN	T	Ex
TUH06	F	79	198	97.6	0.88	AML1	N	*NPM1*, *DNMT3A*, *FLT3*, and *STAG2*	A	Y	N
TUH07	M	52	104	70	0.06	AML4	inv(16), tri(22)	*KIT* and *IKZF1*	F	Y	N
TUH10	F	64	307	93.2	0.09	AML5	N	*NPM1*, *DNMT3A*, *FLT3*, *TET2*, *ATM*, *CBL*, and *CEBPA*	I	Y	N
TUH32	M	33	97	66	0.0	AML4	N	*NPM1*, *DNMT3A*, and *FLT3*	I	Y	N
TUH73	F	50	118	82	N/A	AML2	t(5;11), tri(8)	*FLT3*, *NRAS*, *PTPN11*, and *WT1*	I	Y	N
TUH76	M	74	128	98.1	0.77	AML1	N	*NPM1*, *DNMT3A*, *FLT3*, and *TET2*	I	Y	N
TUH83	F	51	97	76.4	0.36	AML4	N	*NPM1*, *DNMT3A*, and *FLT3*	I	Y	N
TUH84	M	70	281	60	0.25	AML5	N	*NPM1*, *DNMT3A*, and *FLT3*	I	Y	N
TUH93	F	65	177	93.9	0.01	AML1	N	*NPM1*, *FLT3*, *IDH2*, and *BCORL1*	I	Y	N
TUH96	F	54	88	98.9	0.02	AML1	tri(4)	*NPM1*, *FLT3*, *DNMT3A*, *IDH2*, and *DDR2*	I	Y	N
TUH102	M	78	135	86.8	0.04	AML4	N	*NPM1*, *TET2*, and *NRAS*	F	Y	N
TUH109	M	65	94	91	0.16	AML1	N	*NPM1* and *DNMT3A*	F	Y	N
PAR27	M	62	55	94.1	0.24	AML2	t(6;11)	*PTPN11*	A	Y	N

Patients are anonymized and identified by a unique code in the “patient” column. G, gender; M, male; F, female; Age: age in years at time of sample collection; W, white blood cell count (×10^9^/L); B, percentage of blast cells in the sample; LSC, percentage of leukemic stem cells defined by the CD34^+^CD38^−^ phenotype; FAB, French-American-British classification subtypes; s-AML, secondary AML. Karyotype: N, normal karyotype; tri, trisomy; inv(16), inv(16)(p13.1q22) resulting in *CBFB*::*MYH11* fusion; t(5;11), t(5;11)(q35;p15.4) resulting in the *NUP98*::*NSD1* fusion; t(6;11), t(6;11)(q27;q23) corresponding to MLL-AF6 (*KMT2A*::*MLLT4*) fusion; del(5q), del(5)(q13q33). NGS, next-generation sequencing analysis of a panel of genes associated with myeloid neoplasms. ELN 2022 risk classification according to the European LeukemiaNet: F, favorable; I, intermediate; A, adverse; T, serial transplantation assays; Ex, *ex vivo* followed by xenotransplantation assays. Y, yes; N, no.

At diagnosis, leukocyte architecture varied substantially between patients (Figures 1B and S1A; Table S1). While several cases were dominated by CD45_dim_ blasts, others displayed a prominent side-scatter (SSC)-defined mono-like compartment consistent with previously described monocytic differentiation states in AML,^20^ supported by CD14 expression within CD45_dim_ cells (Figure S1B). Serial transplantation progressively reshaped this distribution. The mono-like fraction contracted from *P*_*0*_ to *P*_*2*_, with the most pronounced reductions observed in models that initially carried the highest monocytic burden (Figures 1C, S1C, and S1D). By *P*_*2*_, leukemic populations converged toward a predominantly CD45_dim_ profile, indicating preferential expansion of blast-like output during *in vivo* propagation.

We next examined whether this architectural convergence was accompanied by coordinated changes in surface marker expression.^13^^,^^21^ Despite the shift in output composition, antigen patterns remained largely patient-specific. The proportion of CD34^+^CD38^−^ cells among human CD45_dim_ blasts increased at *P*_*1*_ and returned toward baseline levels at *P*_*2*_ (Figures S1E and S1F; Table S2). Expression of CD371 (CLL1) and CD45RA across CD34^+^CD38^−^ and CD34^+^CD38^+^ compartments remained heterogeneous and did not follow a consistent trajectory across passages (Figures S1G–S1I). CD200 expression within CD45_dim_ blasts showed a decreasing trend across passages, but without a consistent or robust pattern (Figures S1J and S1K). Overall, these data indicate selective and marker-specific adjustments within the blast compartment rather than a uniform phenotypic transition.

In contrast, functional assays revealed a clear and consistent passage effect. In low-input leukemia-initiating cell (LIC) assays^22^ using 10^3^ cells per mouse and a 14-week readout, engraftment was rare with primary samples (1/35 mice, 2.8%) but increased markedly at *P*_*1*_ (19/34, 56%) and *P*_*2*_ (26/32, 81%) under identical conditions (Figures 1D and 1E; Table S3). Classical limiting dilution assays (LDAs)^23^ performed across four cell doses in three independent models confirmed significant enrichment of LSC frequency in *P*_*1*_ and *P*_*2*_ relative to *P*_*0*_, whereas differences between *P*_*1*_ and *P*_*2*_ were modest and varied between patients (Figures 1F and S1L; Table S4).

Taken together, serial xenotransplantation drives convergence toward a CD45_dim_ blast-dominated architecture and markedly increases leukemia-propagating capacity, without consistent remodeling of surface LSC marker expression.

### Leukemic propagation shifts the balance of conserved cell states

**Figure 2:**
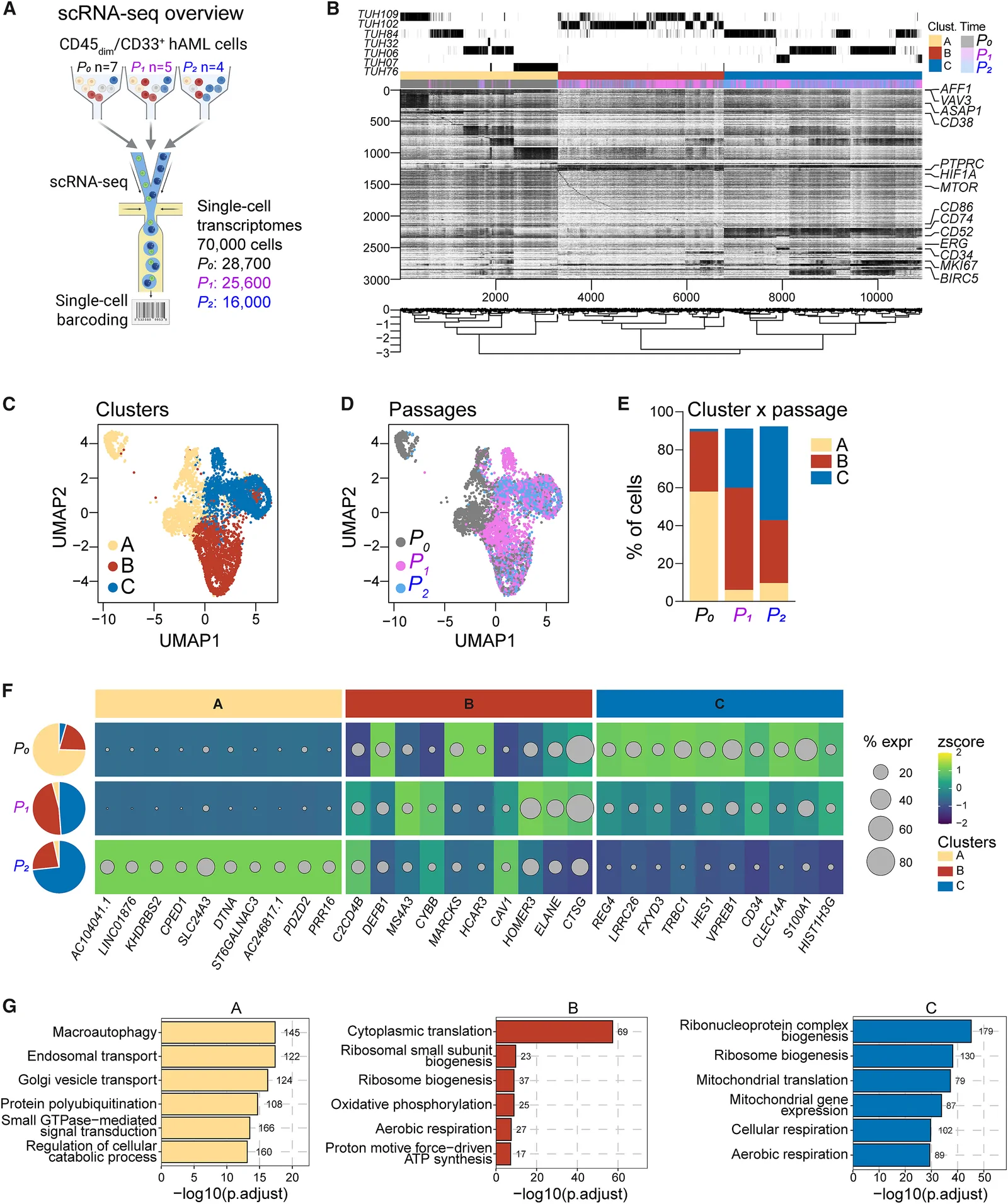
Serial xenotransplantation reshapes the balance between conserved leukemic cell states (A) Single-cell RNA sequencing strategy. CD45_dim_/CD33^+^ AML cells from leukemic samples of different passages were profiled by scRNA-seq. After filtering, ∼70,000 leukemic single-cell transcriptomes were retained. Data include one previously published sample (GEO: GSE178912 and GSM5400788)^24^ processed in parallel with newly generated datasets. (B) Global transcriptional landscape of leukemic cells. Heatmap of gene expression across single cells ordered by hierarchical clustering. Annotation tracks indicate patient identity and xenograft stage. Three major transcriptional populations (A–C) are identified with representative marker genes shown. (C) UMAP projection of leukemic single cells colored by transcriptional cluster, revealing three major populations (A–C). (D) UMAP colored by xenograft stage. (E) Relative contribution of clusters A–C across stages. Stacked bar plots show the proportion of each transcriptional population in *P*_*0*_, *P*_*1*_, and *P*_*2*_ samples. (F) Expression of top marker genes across leukemic clusters and xenograft stages. Rows correspond to clusters across passages, and columns represent marker genes grouped by cluster identity. Dot size indicates the fraction of cells expressing each gene within the cluster, and color represents scaled mean expression (*Z* score). (G) Functional enrichment of gene programs associated with clusters A–C. Selected pathways (Gene Ontology Biological Process, GOBP) are shown with enrichment significance (−log_10_ adjusted *p* value).

Given the increased leukemia-propagating capacity of xenografted cells compared with primary leukemic cells, we next asked whether this is associated with shifts in the balance of leukemic cell states. To address this, we performed single-cell RNA sequencing (scRNA-seq) on CD45_dim_/CD33^+^ human AML cells collected across xenograft stages. After quality filtering, the dataset comprised ∼70,000 leukemic single-cell transcriptomes, including ∼28,700 *P*_*0*_ cells, ∼25,600 *P*_*1*_ cells, and ∼16,000 *P*_*2*_ cells (Figure 2A).

To examine the overall structure of the dataset, we first visualized genome-wide expression patterns across all leukemic cells by using an unsupervised heatmap. This analysis revealed four major cell populations, designated clusters A, B, C, and M (Figure S2A). Comparison with external hematopoietic references^25^ indicated that clusters A–C are enriched in hematopoietic stem and progenitor cell (HSPC) and progenitor features, whereas cluster M corresponds to differentiated myeloid populations (Figure S2B). Analysis of independent AML single-cell datasets^12^^,^^26^ confirmed this pattern, with hematopoietic stem cell (HSC), progenitor, and granulocyte–monocyte progenitor (GMP) programs spanning clusters A–C, while monocyte and dendritic populations are largely confined to cluster M (Figures S2C and S2D). Together, these observations indicate that clusters A–C define an immature leukemic compartment that spans a continuum of transcriptional states.

To better characterize these states, we generated a simplified heatmap based on a random sampling of cells from clusters A–C (Figure 2B). Cluster A expressed activation-associated genes such as *AFF1*, *VAV3*, and *CD38*; cluster B showed higher levels of regulators including *PTPRC* and *HIF1A*, whereas cluster C was enriched for immature and proliferative markers such as *CD34*, *ERG*, and *MKI67*. We next assessed the robustness of the clustering. Silhouette analysis supported the stability of the clustering solution, with cluster A showing the highest separation scores, whereas clusters B and C showed partial overlap, suggesting closely related states (Figure S2E). Uniform manifold approximation and projection (UMAP) visualization further supported this organization, revealing a continuous structure linking clusters A–C and consistent cell distributions across patients (Figures 2C, 2D, S2F, and S2G).

Cells from *P*_*0*_, *P*_*1*_, and *P*_*2*_ occupied largely overlapping transcriptional space within this A‑C compartment, indicating that leukemic propagation did not generate additional transcriptional states. Instead, propagation was associated with shifts in the relative abundance of these populations. *P*_*0*_ samples were predominantly composed of clusters A (63%) and B (35%), whereas *P*_*1*_ and *P*_*2*_ progressively shifted toward clusters B and C, with cluster C becoming dominant at *P*_*2*_ (54%) (Figure 2E). Consistent patterns were observed across our dataset and an independent AML reference dataset^12^ using complementary analytical approaches. Trajectory analysis supports a continuous transcriptional organization of the immature leukemic compartment along pseudotime (Figure S2H).

Gene expression patterns further distinguished these clusters. Expression of representative marker genes confirmed the transcriptional identity of each cluster, with expression patterns consistent across passages (Figure 2F; Table S5). Pathway analysis showed that cluster A was enriched for vesicular and catabolic processes, including macro-autophagy and endosomal transport, whereas clusters B and C were dominated by biosynthetic and energetic programs, including cytoplasmic and mitochondrial translation and respiration-related pathways (Figure 2G). Independent gene sets (Hallmark, Kyoto Encyclopedia of Genes and Genomes [KEGG], and Reactome) gave similar results, consistently highlighting autophagy and endocytic processes in cluster A and translational, metabolic, and cell-cycle-associated programs in clusters B and C (Figure S2I; Table S6).

Together, these analyses indicate that leukemic propagation does not introduce new cellular states but shifts the balance between pre-existing ones, with cells redistributing along a shared transcriptional continuum.

### Progressive non-genetic remodeling is a major driver of leukemic evolution during serial xenotransplantation

**Figure 3:**
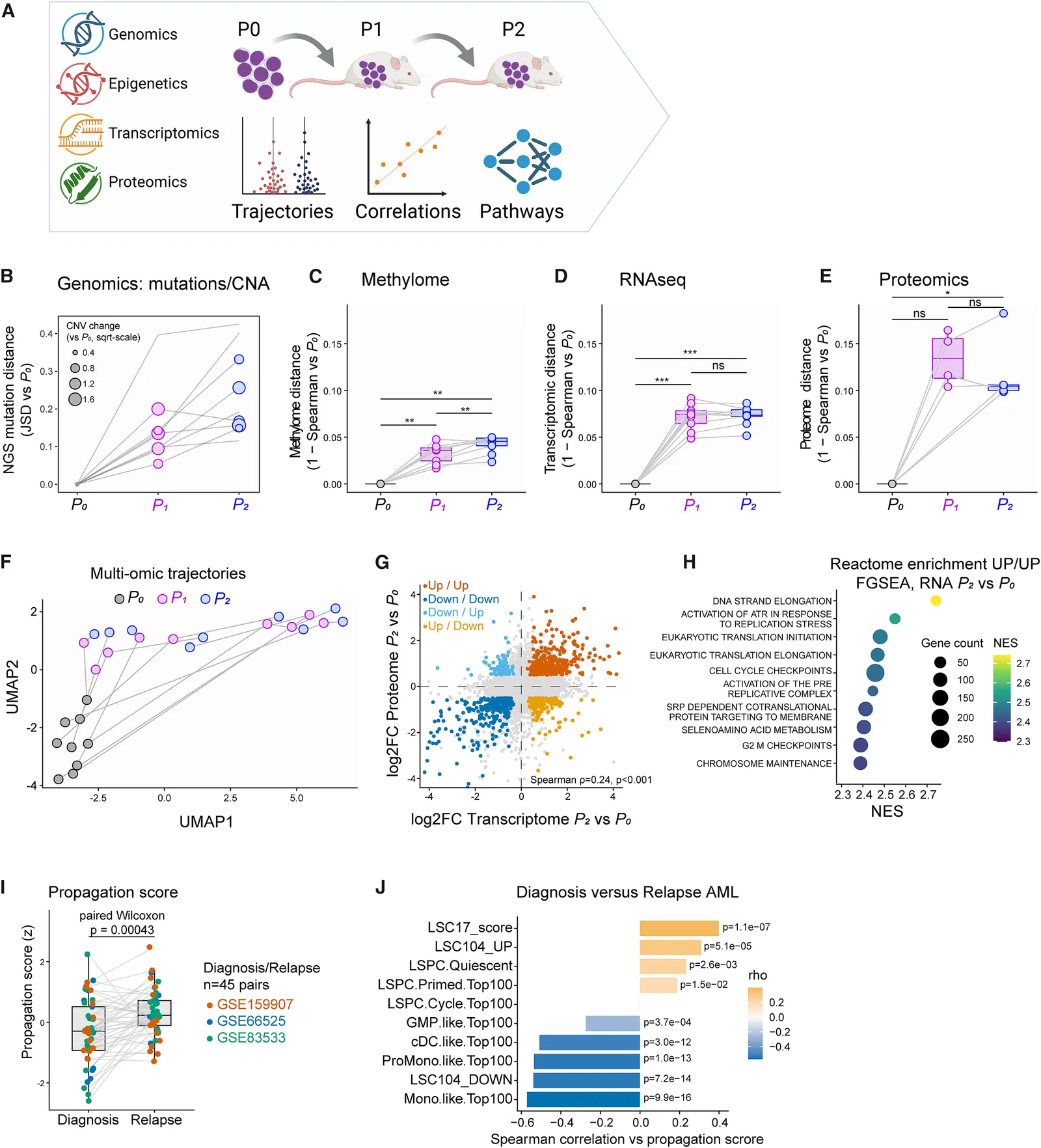
Progressive non-genetic remodeling is a major driver of leukemic evolution during serial xenotransplantation (A) Overview of integrated multi-omics profiling across serial passages, including targeted genomics, DNA methylation, transcriptomics, proteomics, and downstream pathway analyses. All experiments were done in 9 matched trios except for proteomics (*P*_*0*_ = 6, *P*_*1*_ = 5, and *P*_*2*_ = 6 samples). (B) Genetic divergence across passages using Jensen-Shannon distance (JSD) relative to *P*_*0*_ for targeted mutation profiles. Copy number alteration (CNA) change represents the summed magnitude of copy number alterations compared with *P*_*0*_ and is displayed as circle size (square-root-transformed scale). Lines connect matched samples. (C) DNA methylation divergence from *P*_*0*_ was calculated as 1 − Pearson correlation of genome-wide DNA methylation β-values across CpG sites between each xenograft sample and its matched *P*_*0*_ sample. Each dot represents one model at *P*_*0*_, *P*_*1*_, or *P*_*2*_; lines connect matched samples. (D) Transcriptomic distance from *P*_*0*_ was calculated as 1 − Pearson correlation of normalized gene expression values (RNA-seq) across all quantified genes between each xenograft sample and its matched *P*_*0*_ sample. Each dot represents one model; lines connect matched serial samples. (E) Proteomic distance from *P*_*0*_ was calculated as 1 − Pearson correlation of protein abundance values (label-free quantitative mass spectrometry) across all quantified proteins between each xenograft sample and its matched *P*_*0*_ sample. Each dot represents one model; lines connect matched serial samples. (F) UMAP projection of integrated multi-omics profiles. Each point represents one sample; arrows connect serial passages within each model. (G) Gene-wise comparison of RNA and protein log_2_ fold changes (*P*_*2*_ versus *P*_*0*_). Quadrants indicate concordant upregulation, concordant downregulation, or discordant changes. Spearman correlation coefficient is shown. (H) Pathway enrichment for RNA-seq was performed using a rank-based approach (fgsea) applied to log_2_ fold changes across all genes. Proteomic pathway enrichment was performed on differentially abundant proteins (FDR <0.05) using curated gene sets (Reactome). Comparisons were performed between *P*_*2*_ and *P*_*0*_. Dot size reflects gene set size, and color indicates normalized enrichment score (NES). (I) Propagation scores were calculated from genes differentially expressed between propagated xenografts (*P*_*1*_/*P*_*2*_) and primary leukemias (*P*_*0*_), defined as the difference between the average expression of propagation-upregulated and propagation-downregulated genes. The score was applied to independent AML cohorts with matched diagnosis and relapse samples (GEO: GSE159907, GSE66525, and GSE83533; total *n* = 45 pairs). Each dot represents one patient sample; lines connect matched diagnosis and relapse samples. (J) Spearman correlations between the propagation score and established AML cellular-state signatures across patient samples. Stemness-associated programs (LSC17, LSC104, and quiescent or primed LSPC states) showed positive correlations with the propagation score, whereas differentiation-associated signatures (GMP-like, monocyte-like, and dendritic-cell-like states) showed negative correlations. Bars represent Spearman correlation coefficients; corresponding *p* values are indicated. Statistical significance: ns, not significant; ∗*p* < 0.05; ∗∗*p* < 0.01; ∗∗∗*p* < 0.001.

As leukemic propagation was associated with a redistribution of pre-existing cell states, we asked whether these changes are driven by clonal selection or reflect non-genetic remodeling during propagation. To address this, we performed an integrated multi-omics analysis of matched primary (*P*_*0*_), first-passage (*P*_*1*_), and second-passage (*P*_*2*_) AML samples, combining targeted genomics, DNA methylation, transcriptomics, and proteomics (Figure 3A). This framework allowed us to directly compare the magnitude and directionality of genetic versus non-genetic changes across stages of *in vivo* propagation.

At the genomic level, leukemic cells displayed limited evolutionary divergence, consistent with previous comparisons between primary AML samples and xenografted leukemias showing largely preserved clonal architectures.^27^ Targeted sequencing of a 50-gene myeloid panel revealed largely stable mutational landscapes across passages, with only modest shifts in variant allele frequencies for selected lesions such as *FLT3*, *NRAS*, or *WT1* in a subset of models (Figure S3A; Table S7). Quantification of mutational divergence using Jensen-Shannon distance confirmed limited genetic displacement relative to *P*_*0*_, despite inter-patient heterogeneity (Figure 3B). Consistent with this, copy number alterations showed only minor changes during the initial engraftment step, with limited additional changes between *P*_*1*_ and *P*_*2*_ (Figures 3B and S3B). Together, these results indicate that serial xenotransplantation is associated with limited clonal diversification, consistent with the absence of marked subclonal structure at diagnosis in these models.

In contrast to the limited genetic variation observed, epigenetic and transcriptional layers exhibited pronounced and progressive remodeling. Genome-wide DNA methylation profiling revealed a significant divergence from the primary state already at *P*_*1*_, which further increased at *P*_*2*_ (Figure 3C; Table S8). Unsupervised analyses demonstrated that methylation distances from *P*_*0*_ expanded in a stepwise manner across passages, highlighting early and sustained epigenetic reconfiguration. At the promoter level, xenografted samples were characterized by a strong bias toward hypermethylation compared with *P*_*0*_, with effect sizes increasing from *P*_*1*_ to *P*_*2*_ (Figure S3D). Enrichment analysis of differentially methylated promoters identified specific transcription factor target sets, including SOX10-and ZNF-associated networks (Figure S3G), suggesting coordinated rewiring of regulatory circuits rather than stochastic methylation drift.

Transcriptomic profiling mirrored these epigenetic shifts but revealed a distinct temporal pattern. Global RNA expression distances relative to *P*_*0*_ increased sharply at *P*_*1*_, whereas additional divergence between *P*_*1*_ and *P*_*2*_ was comparatively modest (Figure 3D), indicating that most transcriptional reprogramming occurs during the initial *in vivo* expansion. Differential expression and pathway analyses showed that xenografted leukemias downregulated inflammatory and immune-associated programs including TNFα/NF-κB signaling, interferon responses, IL6-JAK-STAT3 signaling, complement and coagulation pathways, while concomitantly activating proliferative and metabolic programs (Figure S3H). These included E2F and MYC target genes, G2/M checkpoint control, DNA repair, oxidative phosphorylation, fatty acid metabolism, glycolysis, and mTORC1 signaling, reflecting a shift toward a highly proliferative and biosynthetically active state.

Integration of proteomic data further refined this view. Proteome-wide distances relative to *P*_*0*_ increased significantly in xenografts but showed limited additional divergence between *P*_*1*_ and *P*_*2*_ (Figure 3E), paralleling transcriptomic dynamics. However, direct coupling between RNA and protein changes was modest, indicating extensive post-transcriptional modulation (Figure 3G). When focusing on concordantly regulated features, jointly upregulated transcripts and proteins converged on core cellular programs, including DNA replication, replication stress responses (ATR signaling), chromosome maintenance, and multiple components of the translation machinery, such as initiation and elongation factors (Figure 3H). These pathways were among the most consistently enriched across xenografts, pointing to a coordinated reinforcement of anabolic and proliferative capacities at multiple regulatory layers.

Finally, projection of patient-level, aggregated multi-omics distance metrics into a low-dimensional space revealed aligned, patient-specific trajectories from *P*_*0*_ toward advanced xenografts, indicating a conserved direction of molecular remodeling across passages despite inter-model variability dominated by non-genetic changes (Figure 3F). Notably, promoter methylation changes did not exhibit a simple inverse relationship with gene expression at the individual gene level (Figure S3E), whereas methylome and transcriptome distances were strongly correlated at the sample level (Figure S3F), supporting a model in which epigenetic remodeling accompanies and stabilizes global transcriptional state transitions.

To place these findings in a clinical context, we analyzed independent AML cohorts with matched diagnosis and relapse samples and derived a propagation score from genes differentially expressed between propagated xenografts and primary leukemias. Across three datasets comprising 45 paired samples (GEO: GSE159907, GSE66525, and GSE83533),^28^^,^^29^^,^^30^ propagation scores were consistently higher at relapse than at diagnosis (Figure 3I). Analysis of paired DNA methylation data from an independent cohort (GEO: GSE159907, *n* = 15 pairs) similarly showed increased propagation-associated methylation scores at relapse (Figure S3J). We next examined how this score relates to established AML cellular-state programs. Higher propagation scores were associated with stemness-related signatures,^25^^,^^26^ including LSC17, LSC104, and quiescent or primed leukemic stem and progenitor cell (LSPC) states, whereas differentiation-associated programs such as GMP-like, monocyte-like, and dendritic-cell-like signatures showed the opposite pattern (Figures 3J and S3K; Tables S9 and S10). These findings indicate that the transcriptional state emerging during xenograft propagation correlates with programs enriched at AML relapse, supporting clinical relevance.

Together, these results indicate that leukemic evolution during serial xenotransplantation is primarily driven by coordinated non-genetic state transitions.

### Integrated multi-omics progression uncovers translational activation and ribosome remodeling during leukemic adaptation

**Figure 4:**
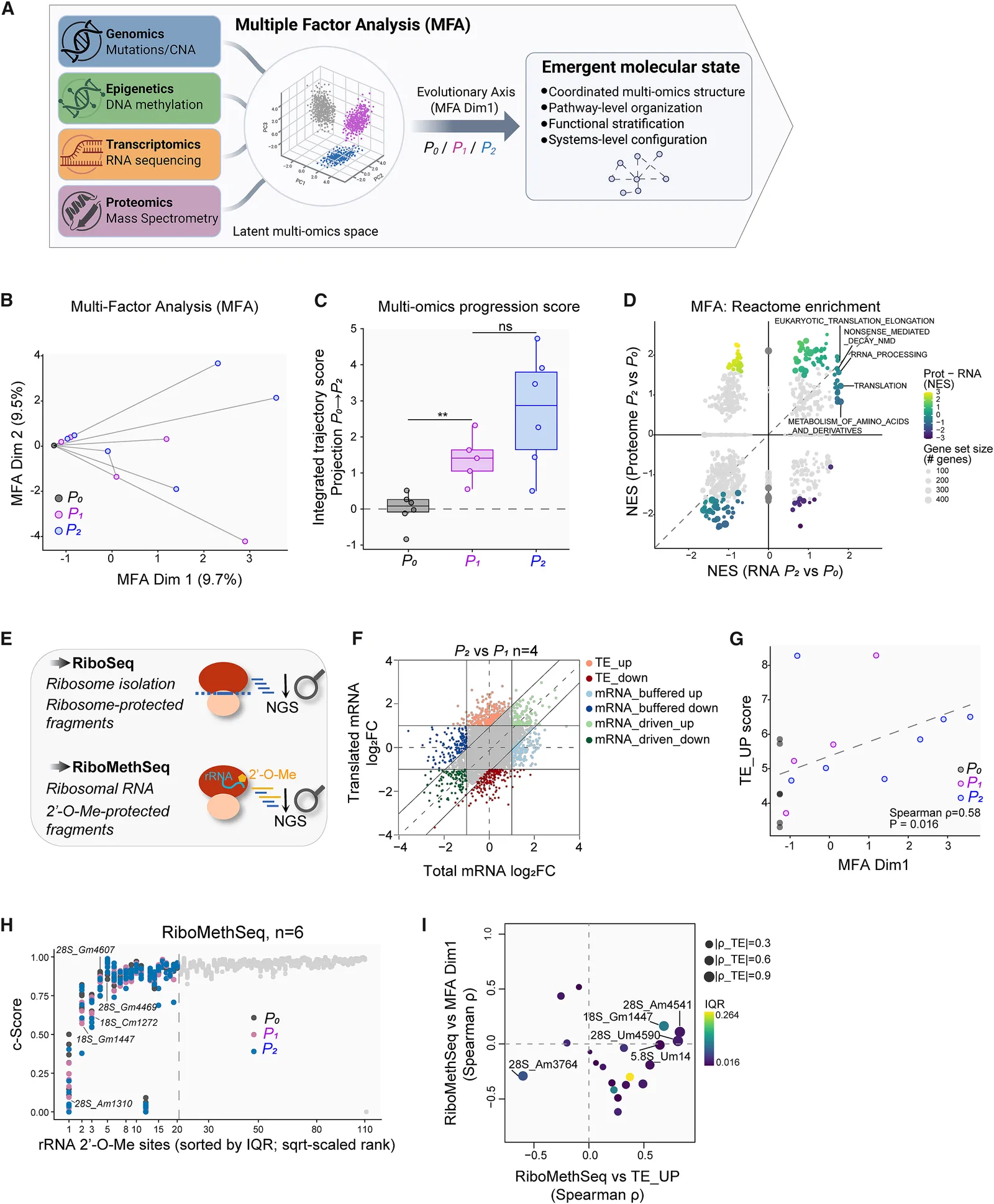
Integrated multi-omics progression uncovers translational activation and ribosome remodeling during leukemic adaptation (A) Schematic of multiple factor analysis (MFA) integrating mutation, copy number, DNA methylation, transcriptomic, and proteomic data to define a unified progression axis. (B) MFA projection of samples across *P*_*0*_, *P*_*1*_, and *P*_*2*_. Lines connect matched serial samples, illustrating directional displacement along Dim1. (C) Quantification of integrated progression scores derived from MFA across stages. (D) RNA-seq analyses used a rank-based approach (fgsea) applied to log_2_ fold changes across all genes, while proteomic enrichment was performed on differentially abundant proteins (FDR <0.05) using curated gene sets (Reactome). Comparisons were performed between *P*_*2*_ and *P*_*0*_. Each point represents one pathway; selected pathways are highlighted. (E) Overview of ribosome profiling (Ribo-Seq, *P*_*1*_*n* = 4, *P*_*2*_*n* = 4) and RiboMethSeq (*n* = 6 matched trios) analyses used to quantify translation efficiency and rRNA 2′-O-methylation. (F) Joint analysis of total mRNA abundance (*x* axis) and ribosome-protected fragments (*y* axis) comparing *P*_*2*_ versus *P*_*1*_. Transcripts are classified as translation efficiency-upregulated (TE_up), TE_down, mRNA-driven, or buffered. (G) Correlation between MFA progression axis (Dim1) and composite TE_up score. (H) Variability of rRNA 2′-O-methylation sites (C-score; *n* = 6 samples), ordered by interquartile range. Selected sites are annotated. (I) Site-specific correlations between rRNA methylation levels and TE_up (left) or MFA Dim1 (right), highlighting preferential association with translational output. Statistical significance: ns: not significant, ∗∗*p* < 0.01.

To investigate how post-transcriptional regulation contributes to leukemic progression during serial xenotransplantation, we integrated genomic, epigenomic, transcriptomic, and proteomic datasets using a multiple factor analysis (MFA) framework (Figure 4A). In this integrated space, primary leukemias clustered distinctly from xenografted samples, while *P*_*1*_ and *P*_*2*_ followed a progressive, patient-specific displacement along the main MFA axis (Dim1), indicating a conserved direction of molecular remodeling across models (Figure 4B). Quantification of this displacement using an integrated progression score revealed a significant shift from *P*_*0*_ to *P*_*1*_, with a further increase at *P*_*2*_, supporting a stepwise and cumulative multi-omics progression rather than a single engraftment-associated transition (Figure 4C; Table S11).

Decomposition of MFA contributions revealed that this trajectory is dominated by non-genetic layers. DNA methylation, transcriptomic, and proteomic signals accounted for the largest share of variance along the progression axis, whereas mutations and copy number alterations contributed minimally (Figures S4A and S4B). This finding is consistent with the limited genetic divergence observed during xenotransplantation and indicates that post-genomic regulation is the principal driver of leukemic adaptation in this setting. At the pathway level, programs that increased along the MFA axis showed strong concordance between RNA and protein layers. Comparison of Hallmark pathway enrichment scores derived independently from transcriptome and proteome data revealed structured alignment of proliferative and metabolic programs across omics, including E2F targets, G2/M checkpoint, MYC targets, and oxidative phosphorylation (Figure S4C). Reactome enrichment further highlighted RNA metabolism and protein synthesis pathways, including translation initiation and elongation, ribosomal RNA processing, nonsense-mediated decay, and amino acid metabolism, as central features of advanced xenografts (Figure 4D; Table S12). These results position translational control as a major component of the integrated molecular trajectory associated with leukemic progression *in vivo*.

To directly interrogate translational regulation within this framework, we performed Ribo-Seq profiling^31^ to quantify changes in translation efficiency between *P*_*1*_ and *P*_*2*_ xenografts (Figure 4E). Joint analysis of total mRNA abundance and ribosome-protected fragments stratified transcripts into distinct regulatory categories, including transcriptionally driven, buffered, and translation-efficiency-regulated groups (Figure 4F; Table S13). While all categories were represented across stages, transcripts exhibiting increased translation efficiency in *P*_*2*_ emerged as a defining feature of advanced xenografts. Transcripts with increased translation efficiency did not converge on a dominant functional pathway, while immune and inflammatory programs showed an inverse association with translation efficiency (Figure S4D). Importantly, a composite translation efficiency-up (TE_up) score derived from Ribo-Seq profiling increased in parallel with the MFA progression axis and showed a positive correlation with Dim1 across samples (Figure 4G). This association indicates that enhanced translational efficiency is not an isolated molecular event, but rather accompanies the global multi-omics progression induced by serial xenotransplantation.

We next examined whether ribosome remodeling accompanies this translational activation. Ribosomal RNA 2′-O-methylation profiling revealed a subset of rRNA sites with high inter-sample variability, several of which exhibited progressive decreases in RiboMethSeq C-score from *P*_*0*_ to xenografts, consistent with reduced methylation at these sites (Figure 4H; Table S14). Several rRNA sites showed statistically significant stage-dependent methylation changes relative to *P*_*0*_, including 18S_Cm1272, 28S_Gm4588, and 18S_Am99 (Figure S4E), supporting selective ribosomal epitranscriptomic remodeling during *in vivo* propagation.^32^ Among these, 18S_Cm1272, 28S_Am1310, and notably, 18S_Gm1447 showed consistent and stage-dependent changes across models. Correlation mapping across variable rRNA sites highlighted a subset of sites whose methylation levels were associated with the TE_up axis, with weaker or more heterogeneous relationships to the global MFA Dim1 axis (Figure 4I). Among these, 18S_Gm1447 showed one of the strongest positive associations with TE_up, correlating site-specific rRNA methylation changes to translational adaptation. Recent work in a 2′-O-methylation-deficient yeast model shows that reduced rRNA methylation alters ribosome structure and fidelity,^33^ suggesting that ribosome remodeling may contribute to selective translational reprogramming during leukemic propagation.

Thus, translational activation and ribosome remodeling constitute central post-transcriptional features of leukemic adaptation during serial xenotransplantation.

### Stage-dependent reorganization of drug response profiles reveals a restricted set of robust vulnerabilities in advanced xenografts

**Figure 5:**
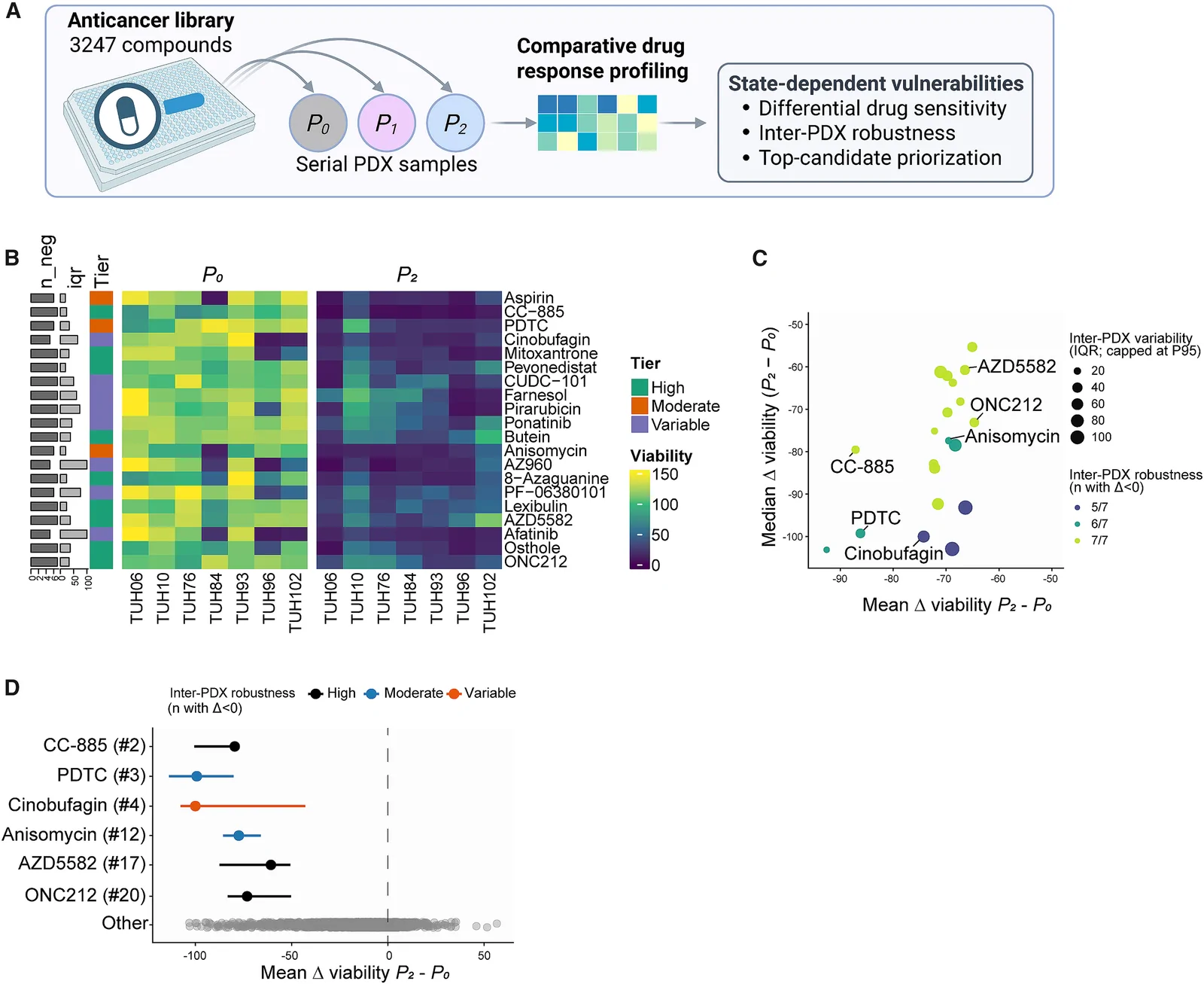
Stage-dependent reorganization of drug response profiles reveals a restricted set of robust vulnerabilities in advanced xenografts (A) Experimental workflow of large-scale drug screening (3,247 compounds) across *P*_*0*_ (*n* = 7), *P*_*1*_ (*n* = 4), and *P*_*2*_ (*n* = 7) AML samples, followed by differential response analysis and prioritization of stage-associated hits. (B) Columns represent individual PDX models at *P*_*0*_ (left block) and *P*_*2*_ (right block). Rows correspond to prioritized compounds. Cell color indicates normalized viability relative to vehicle control, expressed as percentage. n_neg: number of PDX models in which the compound exhibited enhanced activity at *P*_*2*_ compared to *P*_*0*_ (defined as Δ viability [*P*_*2*_ − *P*_*0*_] < 0). IQR, interquartile range of Δ viability (*P*_*2*_ − *P*_*0*_) across models, reflecting inter-model variability of stage-dependent response. Tier indicates compound classification based on combined magnitude and consistency of stage-associated activity, integrating mean Δ viability (*P*_*2*_ − *P*_*0*_), the number of models with enhanced activity at *P*_*2*_ (n_neg), and inter-model dispersion (IQR). To avoid artificial cross-passage clustering, hierarchical clustering of compounds was performed independently within *P*_*0*_ and *P*_*2*_ groups using distance = 1 − Spearman correlation and average linkage. *P*_*1*_ samples were omitted from clustering to maintain consistency with the primary statistical comparison (*P*_*2*_ versus *P*_*0*_). (C) Inter-model consistency of top candidates. Mean versus median differential response (*P*_*2*_ − *P*_*0*_) is shown; dot size and color reflect interquartile range and number of models showing enhanced activity in *P*_*2*_. (D) Prioritization plot showing differential sensitivity (*P*_*2*_ − *P*_*0*_) across compounds. Highlighted compounds represent selected candidates for *in vivo* refinement, with ranks indicating their position among all 3,247 compounds based on mean Δ viability *P*_*2*_ − *P*_*0*_.

To functionally interrogate vulnerabilities emerging during leukemic evolution, we performed a large-scale pharmacological screen across primary and xenografted AML samples using a library of 3,247 compounds enriched for pharmacologically annotated molecules, including FDA-approved drugs, investigational agents, and experimental therapeutics, enabling direct mechanistic interpretation and prioritization of actionable dependencies^34^ (Figure 5A; Tables S15 and S16). Drug exposure resulted in comparable global viability distributions across stages, consistent with a broadly preserved range of *ex vivo* drug sensitivity during serial transplantation (Figures S5A and S5B). We next projected each sample according to its viability pattern across the 3,247-compound library. *P*_*0*_, *P*_*1*_, and *P*_*2*_ samples showed a progressive stage-associated displacement in this pharmacological space, while remaining distinct from leukemia cell lines (Figure S5C).

We next examined whether this reorganization translated into reproducible vulnerabilities. Compounds were ranked by their mean differential activity across matched xenograft pairs. While many compounds showed enhanced activity in *P*_*2*_, most effects were modest or heterogeneous across models. A limited subset, however, showed larger and more concordant effects (Figure S5D). The 20 leading compounds displayed largely concordant patterns across *P*_*2*_-derived samples (Figure 5B). These candidates were further annotated for effect size, inter-PDX robustness, response dispersion, and pharmacological class (Figures 5C, 5D, and S5E).

CC-885 (#2), pyrrolidinedithiocarbamate (PDTC; #3), and cinobufagin (#4) ranked among the strongest and most reproducible candidates. To extend the validation set across distinct pharmacological mechanisms, we also selected anisomycin (#12), AZD5582 (#17), and ONC212 (#20), targeting translation/ribotoxic stress, IAP inhibition, and mitochondrial stress, respectively. We then assessed the six selected candidates across a range of concentrations in independent *P*_*2*_-stage xenografts with distinct overall sensitivity profiles in the initial screen and observed dose-dependent antileukemic activity, supporting their prioritization for *in vivo* evaluation (Figures S5F and S5G).

Together, these analyses provided a rationale for prioritizing six compounds for *in vivo* evaluation.

### Targeting evolution-enriched dependencies suppresses leukemic propagation *in vivo*

**Figure 6:**
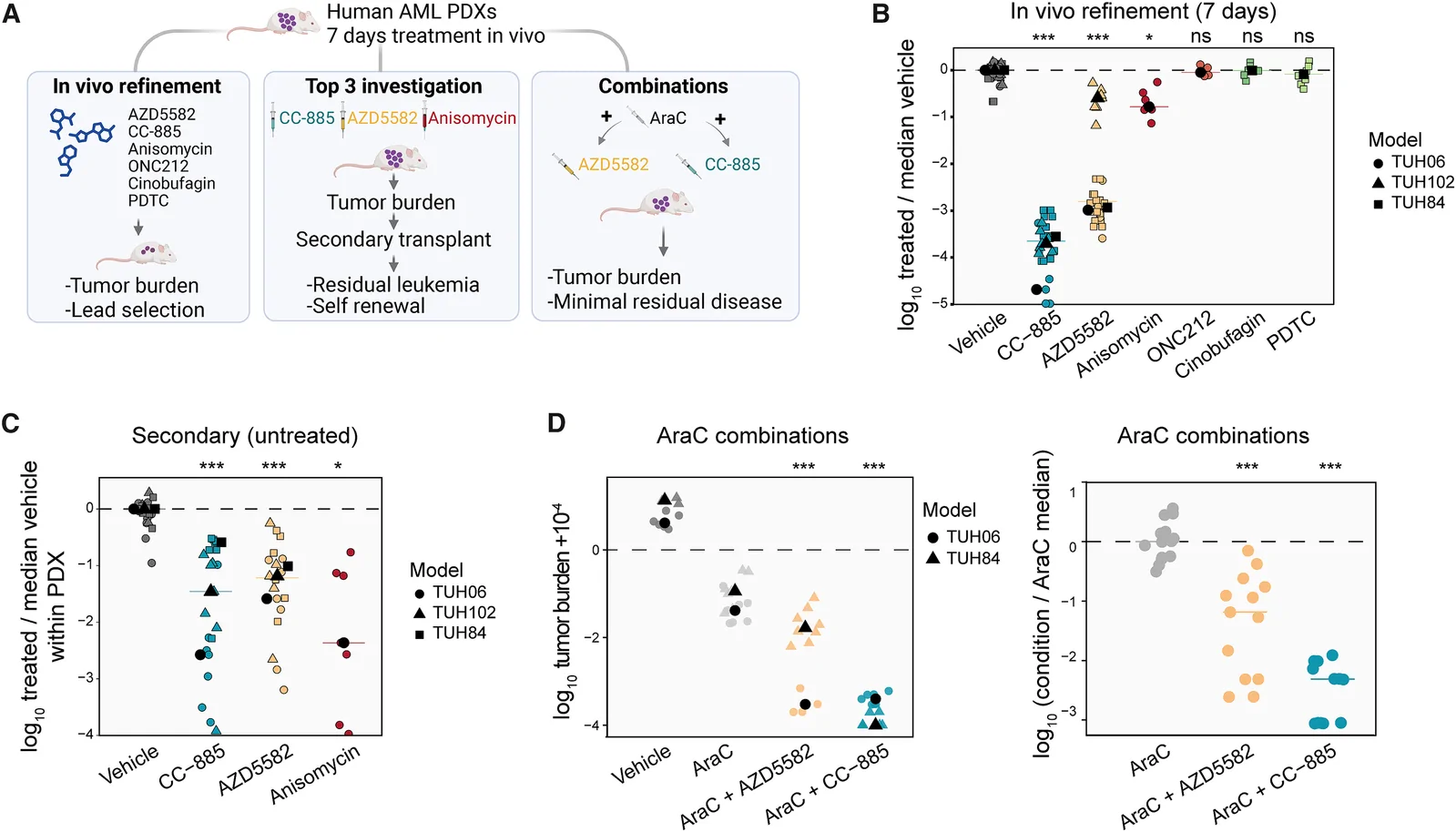
Targeting evolution-enriched dependencies suppresses leukemic propagation *in vivo* (A) Schematic overview of the *in vivo* validation strategy in advanced AML PDXs. PDX-bearing mice (*P*_*2*_, approximately 14 weeks after transplantation) were treated for 7 consecutive days and assessed through three complementary steps: (i) initial *in vivo* refinement to prioritize compounds based on reduction of leukemic burden, (ii) secondary transplantation of viable residual AML cells to measure residual propagation capacity, and (iii) combination studies with cytarabine (Ara-C) to evaluate whether selected agents further reduce disease burden beyond chemotherapy alone. Compounds were administered by intraperitoneal injection at the following doses: AZD5582 (2 mg/kg/day), CC-885 (10 mg/kg/day), anisomycin (10 mg/kg/day), ONC212 (50 mg/kg/day), pyrrolidinedithiocarbamate (PDTC; 50 mg/kg/day), cinobufagin (2 mg/kg/day), and cytarabine (Ara-C; 30 mg/kg/day). (B) *In vivo* refinement screen after 7 days of treatment. Leukemic burden is normalized to the matched vehicle median within each model. AZD5582 and CC-885 were evaluated across three independent PDX models (TUH06, TUH84, and TUH102), anisomycin and ONC212 in TUH06, and cinobufagin and PDTC in TUH84. (C) Residual leukemic propagation capacity measured by secondary transplantation of viable AML cells harvested after the 7-day treatment period. Cells were normalized for input and transplanted into untreated secondary recipients. Engraftment is shown as log_10_(treated/median vehicle within each PDX). (D) Combination studies with Ara-C in TUH06 and TUH84. Left, leukemic burden after treatment with vehicle, Ara-C, Ara-C + AZD5582, or Ara-C + CC-885, shown as log_10_ tumor burden. Right, the same data normalized to the Ara-C median within each PDX. Each dot represents one mouse. Black horizontal bars indicate group medians. Black symbols indicate medians for each PDX model separately. For normalized analyses, values were compared against 0 using two-sided Wilcoxon tests with Holm correction within the relevant comparison families. ns, not significant; ∗*p* < 0.05; ∗∗*p* < 0.01; ∗∗∗*p* < 0.001.

To test whether vulnerabilities emerging during leukemic progression remain actionable *in vivo*, we established a 7-day treatment framework in advanced AML PDXs combining three readouts: on-treatment leukemic burden, secondary transplantation to assess residual propagation capacity, and combination studies with Ara-C (Figure 6A). Treatments were initiated in established *P*_*2*_ xenografts after confirmation of human AML engraftment. In this refinement phase, several compounds were evaluated, and CC-885, AZD5582, and anisomycin each induced significant cytoreduction (Figure 6B).

Additional measurements helped interpret these early effects. In TUH06, CC-885 and anisomycin increased DRAQ7 positivity, whereas AZD5582 showed a weaker change on this short-term cell death readout (Figure S6A). Over the same period, AZD5582 was associated with reduced body weight and lower area under the curve (AUC), while CC-885 and anisomycin had more limited effects on these parameters (Figures S6B and S6C). Murine bone marrow cellularity also showed distinct patterns across treatments, increasing with AZD5582 and more clearly with CC-885, but decreasing with anisomycin (Figure S6D). The apparent preservation of murine hematopoiesis with CC-885 is consistent with its limited engagement of murine cereblon,^35^ indicating that the three active compounds differed not only in efficacy but also in their effects on host tissues.

We next examined whether these short-term responses translated into changes in leukemia-propagating capacity. Viable human AML cells were recovered after treatment, normalized for input, and transplanted into untreated secondary recipients. Prior exposure to CC-885, AZD5582, or anisomycin reduced secondary engraftment compared with vehicle controls (Figure 6C), indicating that even brief *in vivo* treatment impairs the ability of residual cells to reinitiate disease. We integrated these results with effects on normal human hematopoietic progenitors. In methylcellulose assays, CC-885 and anisomycin markedly impaired CD34^+^ colony formation, whereas AZD5582 showed a more modest effect without a clear dose-response (Figure S6E). The comparatively limited cytoreductive activity of anisomycin, together with its impact on normal progenitors, argued against its prioritization, while CC-885 and AZD5582 were retained for subsequent *in vivo* evaluation.

CC-885 induces CRBN-dependent degradation of GSPT1, whereas AZD5582 targets IAP-family proteins.^16^^,^^17^ Consistent with this, matched *P*_*0*_/*P*_*2*_ immunoblots across independent PDX models showed increased expression of CRBN, GSPT1/2, XIAP, and cIAP1 in *P*_*2*_ samples (Figure S6F). To extend these observations to primary disease, AML samples were exposed *ex vivo* prior to transplantation. Although short-term exposure caused limited immediate cytotoxicity, prior treatment with CC-885 or AZD5582 reduced subsequent *in vivo* engraftment (Figures S6G and S6H; Table S17), indicating an effect on leukemia-initiating capacity not captured by short-term viability alone.

We next examined combination strategies with Ara-C. In independent PDX models, Ara-C reduced leukemic burden,^22^^,^^36^ and the addition of either CC-885 or AZD5582 further decreased disease levels (Figure 6D, left). When normalized to Ara-C, both combinations retained additional activity, confirming cytoreduction beyond chemotherapy alone (Figure 6D, right). These combinations showed distinct effects on host tissues. Adding AZD5582 to Ara-C increased body-weight loss and reduced AUC, whereas CC-885 did not worsen these parameters (Figures S6I and S6J). In parallel, murine bone marrow cellularity remained reduced with Ara-C and AZD5582 but was preserved with CC-885 (Figure S6K).

Together, these results show that vulnerabilities emerging during leukemic progression can be targeted *in vivo* to reduce both leukemic burden and the capacity of residual cells to sustain disease.

## Discussion

Serial xenotransplantation is widely used to define leukemia-initiating capacity, yet the molecular programs selected during this process remain incompletely characterized. By combining longitudinal functional assays with single-cell transcriptomics, multi-omics profiling, ribosome analysis, and large-scale pharmacological screening across matched PDX stages, we followed how leukemic cells evolve across successive passages *in vivo*.

Across passages, LIC frequency increased markedly, whereas mutational and copy number landscapes remained largely stable, indicating that enhanced propagation capacity is associated with shifts in the relative abundance of pre-existing cell states rather than genetic diversification. Single-cell analyses identified a restricted set of leukemic states shared across patients, consistent with previously described AML hierarchies.^12^^,^^25^ Stemness-associated programs were distributed across overlapping immature states, supporting a continuum organization, while serial transplantation reshaped the relative abundance of pre-existing populations, with enrichment of proliferative and biosynthetic programs.

This increase in propagation was not accompanied by consistent shifts in canonical surface LSC markers, consistent with studies comparing diagnosis and post-chemotherapy samples in patients with AML that similarly report increased LSC frequency together with greater phenotypic diversity after treatment.^37^ AML-associated antigens, including CLL1/CD371, CD45RA, CD123, CD47, TIM3, and CD99, do not define a uniform LSC phenotype and are instead used in combination to distinguish immature leukemic cells from normal stem/progenitor populations.^38^^,^^39^^,^^40^ Together, these findings indicate that leukemic propagation is associated with changes in the relative abundance of pre-existing cell states rather than the emergence of a distinct phenotypic compartment.

Multi-omics analyses showed coordinated epigenetic and transcriptional remodeling. DNA methylation divergence appeared early and increased across passages, while transcriptomic and proteomic changes consistently pointed to proliferation, DNA replication, and metabolic pathways. Despite modest RNA-protein concordance, integration across molecular layers revealed a conserved progression axis shared across models and aligned with the transcriptional continuum observed at single-cell level. These observations define a non-genetic trajectory of leukemic progression linking redistribution of pre-existing cell states to functional reconstitution capacity. Consistent with this, propagation-associated molecular features were enriched in independent diagnosis-relapse AML cohorts.

Translational control emerged as a central trait of advanced xenografts. Increased translation efficiency was accompanied by remodeling of specific rRNA 2′-O-methylation sites, whose levels correlated with translational output. Clusters enriched for biosynthetic and mitochondrial programs also showed strong signatures of ribosome biogenesis and both cytoplasmic and mitochondrial translation, highlighting translational activity as a key feature of propagation, consistent with recent work linking rRNA methylation dynamics to stemness programs in AML.^32^

Drug-response profiles also evolved across passages, with advanced xenografts converging on a restricted set of vulnerabilities. Compounds targeting translation, including CRBN-dependent degradation of GSPT1 by CC-885 and ribosome-directed anisomycin,^41^ as well as survival signaling through IAP antagonism with AZD5582, emerged as prominent hits. Even short *in vivo* exposure was sufficient to reduce leukemia-propagating capacity, indicating effects beyond immediate cytoreduction.

Integrating these responses with host-related readouts provided additional context for compound prioritization. CC-885 and AZD5582 showed strong antileukemic activity, whereas anisomycin combined weaker cytoreduction with less favorable effects on murine hematopoiesis and human progenitors. AZD5582 displayed reduced tolerability in mice but only modest effects on human progenitors, suggesting differential host sensitivity. In contrast, CC-885 showed the most pronounced cytoreductive activity, supporting its prioritization despite its toxicity against normal human hematopoietic progenitors.

GSPT1 degraders and IAP antagonists have previously shown anti-leukemic effects in preclinical models.^35^^,^^42^^,^^43^ Early-phase clinical studies of the GSPT1 degrader CC-90009 have reported measurable activity with manageable hematologic toxicity, despite frequent myelosuppression (NCT02848001), supporting the feasibility of targeting this pathway in patients. In this context, both CC-885 and AZD5582 further reduced leukemic burden when combined with Ara-C^44^ in our model, suggesting that such combinations may enhance therapeutic efficacy.

In summary, serial xenotransplantation reveals a largely non-genetic trajectory of leukemic evolution marked by coordinated epigenetic and translational remodeling, redistribution of conserved cell states, and the emergence of stage-specific therapeutic dependencies. Targeting these programs reduces leukemic burden and limits disease reconstitution *in vivo*, highlighting actionable vulnerabilities within propagation-competent leukemic populations.

### Limitations of the study

Xenotransplantation into immunodeficient mice exposes leukemic cells to conditions that differ from those in patients and may favor highly proliferative or metabolically active states. The trajectory described here, therefore, reflects adaptation to this setting and may not fully capture the biology of relapse.

Secondary transplantation shows whether leukemic cells can reinitiate disease but does not identify which cells are responsible or how they change over time. Addressing this will require longitudinal patient samples to follow clonal and cellular dynamics during disease evolution.

Finally, the therapeutic window of the identified vulnerabilities cannot be defined in this study. The experimental framework was designed to uncover functional dependencies rather than to support full preclinical evaluation of candidate compounds. The toxicity-related observations should therefore be considered preliminary, and a rigorous assessment of safety and selectivity will require dedicated preclinical models beyond the scope of this work.

## Resource availability

### Lead contact

The lead contact for this work is Jerome Tamburini (jerome.tamburinibonnefoy@unige.ch).

### Materials availability

This study did not generate unique reagents.

### Data and code availability

• All data associated with this study are present in the paper, the supplemental information, or the source data files. Datasets generated in this study have been deposited in public repositories and are publicly available as follows: DNA methylation EPIC array, GEO: GSE330065; bulk RNA-seq, GEO: GSE329505; single-cell RNA-seq, GEO: GSE329946; Ribo-Seq, GEO: GSE330064; and proteomics, PRIDE/ProteomeXchange PRIDE: PXD078079.
• This paper does not report original custom computer code. Data were analyzed using publicly available software packages and standard workflows, as described in the STAR Methods and listed in the key resources table where applicable.
• Any additional information required to reanalyze the data reported in this work paper is available from the lead contact upon request.

## Acknowledgments

We thank Professor Pierre-Yves Dietrich (Geneva, Switzerland) for having facilitated the development of this study. We thank the Flow Cytometry, Bioimaging, Genomics (iGE3); Proteomics, Reader Assay Development and Screening (READS); RNA to Proteins (BioCode); and Zootechnie Core Facilities of the University of Geneva for technical support. We thank the Ribosomics facility (Fleur Bourdelais) at the Cancer Research Center of Lyon (France) for technical support for the RiboMethSeq analyses. This work was supported by grants from the 10.13039/501100006388Geneva University Hospitals Private Foundation, the Dr. Henri Dubois-Ferrière Dinu Lipatti Foundation, the Promex Foundation for Research, the Nova Foundation, and the Ligue Genevoise contre le Cancer. This work was also supported by grants from the Translational Research Center for Hemato-Oncology (10.13039/501100006389University of Geneva, 10.13039/501100020971Faculty of Medicine, Geneva, Switzerland) funded by the Copley May Foundation, the 10.13039/501100008597MEDIC Foundation, and the Pastré Foundation. C.P.-C. is funded by a Swiss National Foundation grant (SNF 219260), and E.B. is funded by a 10.13039/501100004361Swiss Cancer League grant (KFS-6032-02-2024). F.C. and J.-J.D. are recipients of the Agence Nationale pour la Recherche CryoMeth ANR-22-CE11-0017 grant.

## Author contributions

C.L. designed and performed most of the experiments, performed formal analyses, contributed to data visualization, and revised the manuscript. P.A. contributed to the design and analyzed the methylome, RNA-seq, and scRNA-seq data. S.M. carried out *in vitro* and *in vivo* experiments and performed formal analyses. A.S.G. analyzed flow cytometry data and assisted in data visualization. E.B. analyzed RNA-seq and scRNA-seq data and contributed to data visualization. I.B. contributed to transcriptomic and proteomic data analysis. C.P.-C. performed and analyzed selected flow cytometry experiments. R.B. contributed to formal analysis and revisions of the manuscript. T.A.M. and O.K. analyzed the DNA sequencing data. A.S. contributed to the PDX repository. N.C. and F.V. performed and analyzed flow cytometry experiments. C.R. and V.M.-D.M. treated patients and participated in data collection. P.S. participated in data analysis and revision of the manuscript. F.C. and J.-J.D. performed and analyzed RiboMethSeq experiments. J.-E.S. established the Human Immune Monitoring Initiative Program (HIMIP) collection, participated in data collection, and revised the manuscript. P.T. designed and supervised RNA-seq and scRNA-seq analyses, oversaw computational workflows, and secured funding. J.T. conceived and supervised the project, performed analyses and data visualization, secured funding, and drafted and revised the manuscript. All authors revised and approved the final manuscript.

## Declaration of interests

The authors declare no competing interests.

## STAR★Methods

### Key resources table

**Table:** 

REAGENT or RESOURCE	SOURCE	IDENTIFIER
**Antibodies**		

FITC mouse anti-human CD45	BD Biosciences	Cat# 555482; RRID:AB_395874
APC mouse anti-human CD45	BD Biosciences	Cat# 555485; RRID:AB_398600
PerCP-Cy5.5 mouse anti-human CD33	BD Biosciences	Cat# 333146; RRID:AB_2868647
PE mouse anti-human CD33	BD Biosciences	Cat# 555450; RRID:AB_395843
FITC mouse anti-human CD33	BD Biosciences	Cat# 555626; RRID:AB_395992
BV421 mouse anti-human CD34	BD Horizon	Cat# 562577; RRID:AB_2687922
PE mouse anti-human CD34	BD Biosciences	Cat# 555822; RRID:AB_396151
APC mouse anti-human CD38	BD Biosciences	Cat# 555462; RRID:AB_398599
PE-Cy7 mouse anti-human CD38	BD Biosciences	Cat# 335790; RRID:AB_399969
PE rat anti-mouse CD45	BD Biosciences	Cat# 553081; RRID:AB_394611
PE mouse anti-mouse CD45.1	BD Biosciences	Cat# 553776; RRID:AB_395044
Rabbit pAb anti-GSPT1	Invitrogen	Cat# PA5-62621; RRID:AB_2642244
Rabbit pAb anti-GSPT2	Invitrogen	Cat# PA5-60824; RRID:AB_2642245
Rabbit mAb anti-CRBN (D4K2F)	Cell Signaling	Cat# 71810; RRID:AB_2799810
Rabbit mAb anti-XIAP (D2E1)	Cell Signaling	Cat# 2045; RRID:AB_2214866
Rabbit mAb anti-*c*-IAP1 (D5L9G)	Cell Signaling	Cat# 7065; RRID:AB_10890862
Mouse mAb anti-β-actin	Sigma-Aldrich	Cat# A2228; RRID:AB_476697
HRP goat anti-mouse IgG (H + L)	Thermo Fisher	Cat# 31430; RRID:AB_228307
HRP goat anti-rabbit IgG (H + L)	Thermo Fisher	Cat# 31460; RRID:AB_228341

**Biological samples**		

Primary AML specimens	HIMIP Biobank	DC 2008–307, Toulouse
Primary AML specimens	Cochin Biobank	DC 2015-08-11, Paris

**Chemicals, reagents and recombinant proteins**		

CC-885	MedChemExpress	Cat# HY-101473
AZD5582	MedChemExpress	Cat# HY-100567
Cinobufagin	MedChemExpress	Cat# HY-N0011
Aspirin	MedChemExpress	Cat# HY-B0166
GSK3145095	MedChemExpress	Cat# HY-111082
2,6-Pyridinedicarbothioic acid (PDTC)	MedChemExpress	Cat# HY-B0383
Cytarabine (Ara-C)	MedChemExpress	Cat# HY-B0570
Busulfan (Busilvex)	Sigma-Aldrich	Cat# B2635
β-Mercaptoethanol	Sigma-Aldrich	Cat# M6250
Recombinant human SCF	PeproTech	Cat# 300-07
Recombinant human FLT3L	PeproTech	Cat# 300-19
Recombinant human TPO	PeproTech	Cat# 300-18
Recombinant human IL-3	PeproTech	Cat# 200-03
Recombinant human IL-6	PeproTech	Cat# 200-06
Recombinant human G-CSF	PeproTech	Cat# 300-23
BIT 9500 Serum Substitute	StemCell Technologies	Cat# 09500
Turbo DNase I	Roche	Cat# 04716728001
Cycloheximide	Sigma	Cat# C7698
Protease inhibitors	Roche	Cat# 04693132001
Dulbecco’s modified Eagle’s medium (DMEM)	Thermo-Fisher Scientific	Cat# 61965
Fetal calf serum (FCS)	Eurobio Scientific	Cat# CVFSVF00-01
Anti-cancer compound library	MedChemExpress	HY-L025

**Critical commercial assays and kits**		

EZ DNA Methylation Kit	Zymo Research	Cat# D5001
DNA/RNA Mini Kit	Qiagen	Cat# 80204
Infinium MethylationEPIC BeadChip	Illumina	Cat# WG-317-1001
TruSeq Stranded mRNA Library Prep	Illumina	Cat# 20020594
AmpliSeq Myeloid Panel	Illumina	Cat# A31445
Chromium Next GEM Single Cell 3′ v3.1	10x Genomics	Cat# 1000121
SP3-iST Proteomics Kit	PreOmics	Cat# P.O.00027
RiboCop rRNA Depletion Kit V2	Lexogen	Cat# 144
Direct-zol RNA MiniPrep Plus	Zymo Research	Cat# R2070
ATPlite Luminescence Assay	PerkinElmer	Cat# 6016943
GeneChip WT Plus Reagent Kit	Thermo Fisher	Cat# 902280
Fixable Viability Stain 510	BD Biosciences	Cat# 564406

**Deposited data**		

Bulk RNA-seq	This paper	GEO: GSE329505
Single-cell RNA-seq	This paper	GEO: GSE329946
Ribo-seq	This paper	GEO: GSE330064
DNA methylation (EPIC)	This paper	GEO: GSE330065
DIA-MS Proteomics	This paper	PRIDE: PXD078079

**Experimental models: Cell lines**		

MOLM-14	Not trackable, present in the laboratory for years.	Identified by PCR-single-locus technology (Promega, PowerPlex21 PCR Kit, Eurofins Genomics)
OCI-AML2	Not trackable, present in the laboratory for years.	Identified by PCR-single-locus technology (Promega, PowerPlex21 PCR Kit, Eurofins Genomics)

**Experimental models: Organisms/strains**		

NOD/LtSz-SCID/IL2Rγnull (NSG)	Jackson Laboratory	RRID:IMSR_JAX:001303

**Software and algorithms**		

FlowJo v10.9	BD Biosciences	RRID:SCR_008520
Cell Ranger v7.2	10x Genomics	RRID:SCR_017344
Seurat v5.0	Satija Lab	RRID:SCR_016341
STAR v2.6.1day	Dobin et al.	RRID:SCR_004463
Salmon v1.5.2	Patro et al.	RRID:SCR_017036
DESeq2 v1.34.0	Love et al.	RRID:SCR_015687
limma v3.50.0	Ritchie et al.	RRID:SCR_010943
SeSAMe v1.14.2	Zhou et al.	Kilham^45^
nclust v2.5.0	CNRS	https://doi.org/10.5281/zenodo.8036406
clusterProfiler v4.10.1	Yu et al.	RRID:SCR_016884
Spectronaut v19	Biognosys	N/A
RiboSeQC v2.0	Calviello et al.	Ahmed^46^
rRMSAnalyzer	GitHub	https://github.com/RibosomeCRCL/rRMSAnalyzer
Ion Reporter v5.18	Thermo Fisher	N/A
TSVC v5.6	Thermo Fisher	N/A
Polydiag Suite	Université Paris Cité	https://bioinfo.biomedicale.parisdescartes.fr/
NextGENe v2.4	Softgenetics	N/A
GenomeStudio	Illumina	N/A
Trimmomatic	Bolger et al.	Bolger^47^
Bowtie 3	Ben Langmead	RRID:SCR_016368
R v4.4.1	R Core Team	https://www.r-project.org/
tidyverse v2.0.0	Wickham et al.	https://doi.org/10.21105/joss.01686
ggplot2 v3.5.0	Wickham H.	ISBN:978-3-319-24277-4
ComplexHeatmap v2.16.0	Gu et al.	https://doi.org/10.1093/bioinformatics/btw313
FactoMineR v2.8	Le et al.	https://doi.org/10.18637/jss.v025.i01
fgsea v1.26.0	Korotkevich et al.	https://doi.org/10.1093/bioinformatics/btaa802
msigdbr v7.5.1	Dolgalev	CRAN package
ReactomePA v1.44.0	Yu & He	https://doi.org/10.1039/C5MB00663E
cluster v2.1.4	R package	https://cran.r-project.org/package=cluster
uwot v0.1.16	R package	https://cran.r-project.org/package=uwot
slingshot v2.8.0	Bioconductor	RRID:SCR_017012

**Other instruments**		

CytoFLEX Flow Cytometer	Beckman Coulter	RRID:SCR_025067
Astrios EQ Cell Sorter	Beckman Coulter	RRID:SCR_022169
NextSeq 500 Sequencer	Illumina	RRID:SCR_014983
NovaSeq 6000 Sequencer	Illumina	RRID:SCR_016387
Orbitrap Fusion Lumos MS	Thermo Fisher Scientific	RRID:SCR_020562
Vanquish *Neo* UHPLC	Thermo Fisher Scientific	N/A
SpectraMax L384 Reader	Molecular Devices	RRID:SCR_018598
iScan System	Illumina	RRID SCR_016388
Agilent 2200 TapeStation	Agilent	RRID SCR_014994

### Experimental model and study participant details

#### Human subjects

Primary AML specimens (*n* = 13) were obtained from patients with a confirmed diagnosis of AML after written informed consent, in accordance with the Declaration of Helsinki. Samples originated from two French collections. Toulouse samples were obtained through the Human Immune Monitoring Initiative Program (HIMIP) collection (BB-0033-00060), officially registered with the French Ministry of Higher Education and Research (DC 2008–307, collection 1). Before sample acquisition, a transfer agreement (AC 2008–129) was signed after approval by the Comité de Protection des Personnes Sud-Ouest et Outremer II (ethical committee). Paris samples were obtained under approval from IRB Ile de France II (2015-08-11-DC). The AML patient database was declared to the Commission Nationale de l'Informatique et des Libertés (CNIL) under the MR-004 French reference methodology (registration number 2214849v0). All samples were de-identified prior to analysis.

Patient-level clinicopathologic information, including age, gender, diagnosis, disease status, previous treatment, sample source, and relevant clinical annotations, is provided in Table 1 and in the corresponding source data. Race, ethnicity, and ancestry were not collected or reported. Primary AML samples were not prospectively allocated to clinical intervention groups. For experimental analyses, samples were analyzed according to the study design and sample availability across primary AML specimens and xenografts, with assay-specific sample sizes reported in the relevant figures, tables, and source data.

Previously published de-identified human AML datasets used for diagnosis–relapse comparisons were obtained from public repositories and analyzed according to the disease-stage annotations provided by the original studies. No additional human participant recruitment or intervention was performed for these analyses.

#### Mouse models

Adult 6–8-week-old male and female NOD/LtSz-SCID/IL2Rγ-null mice (NSG; RRID: IMSR_JAX:001303) were housed under specific pathogen-free conditions at the University of Geneva animal facility. All animal procedures conformed to ARRIVE guidelines and were approved by the Geneva Cantonal Authority under protocols GE/123/19 and GE/166.

For patient-derived xenograft experiments, NSG mice were conditioned with busulfan (20 mg/kg, intraperitoneally) and injected intravenously through the tail vein with 0.5–2 × 10^6^ viable primary or xenograft-derived AML cells. Bone marrow engraftment was assessed 14–24 weeks after transplantation by flow cytometry for human hCD45^+^ hCD33^+^ leukemic cells.

#### Cell culture

Primary AML and xenograft-derived AML cells were not maintained as long-term cell lines. For *ex vivo* drug screening and dose-response assays, bulk xenograft samples with high leukemic infiltration were used directly when human leukemic infiltration exceeded 70%, or after murine cell depletion using anti-mouse CD45 magnetic separation when infiltration was lower. Cells were cultured in IMDM/BIT medium supplemented with recombinant human SCF, FLT3L, TPO, IL-3, IL-6, and G-CSF (50/50/25/10/10/10 ng/mL) and 50 μM β-mercaptoethanol.

MOLM-14 and OCI-AML2 human AML cell lines were cultured in Dulbecco’s modified Eagle’s medium (DMEM) supplemented with 10% fetal calf serum at 37°C in a humidified incubator with 5% CO_2_. Cell line identity was authenticated by PCR single-locus profiling using the PowerPlex 21 PCR kit (Promega; Eurofins Genomics). Cells were routinely tested for mycoplasma contamination and were negative.

#### Sex and gender considerations

Patient gender and mouse sex were recorded as descriptive variables. The study was not powered to assess the influence of patient gender or mouse sex on the reported results, and gender- or sex-stratified analyses were therefore not performed.

### Method details

#### Flow cytometry and cell sorting

Cells were stained with the antibodies listed in key resources table and analyzed on a CytoFLEX (Beckman Coulter). hCD45^+^ hCD33^+^ populations were FACS-purified on an Astrios cell sorter (Beckman Coulter). Data were processed in FlowJo v10.9 (RRID:SCR_008520). All downstream omics analyses (single-cell RNA-seq, bulk RNA-seq, DNA methylation, Ribo-seq, RiboMeth-seq and proteomics) were performed on FACS-purified human AML blasts defined as hCD45^+^ hCD33^+^ cells.

#### Limiting dilution assays (LDA) and statistical modeling

Limiting dilution assays were performed by transplanting graded numbers of leukemia cells (10^2^–10^6^ cells) into NSG recipient mice (four mice per dose). Engraftment was scored as a binary outcome (engrafted vs. non-engrafted) based on the presence of ≥0.1% hCD45^+^ human leukemic cells in bone marrow at endpoint. LSC frequency was estimated using generalized linear models (GLMs) with binomial error distribution and complementary log–log (cloglog) link function, appropriate for extreme limiting dilution experiments. In this framework, the probability of engraftment is modeled as: log(−log(1 − p)) = log(dose) + β, where p is the probability of engraftment at a given cell dose. The LSC frequency (f) is derived from the intercept parameter of the fitted model, and is reported as 1/f (cells per LSC). Confidence intervals were calculated using profile likelihood estimation. Differences in LSC frequency across transplantation stages were assessed using likelihood ratio tests comparing nested GLM models. An overall test including all mice (“GLM all mice”) was performed to evaluate stage-dependent differences in engraftment probability across the full dataset.

#### Single-cell RNA-seq

##### Data acquisition

Single-cell RNA-seq was performed and analyzed as reported.^48^ FACS-purified viable hCD45^+^ hCD33^+^ cells were resuspended at 1.5 × 10^3^ cells/μL in 0.04% BSA/PBS and processed with Chromium Next GEM 3′ v3.1 chemistry (10x Genomics). Libraries (200–700 bp) were sequenced on a NextSeq 500 to a depth of ∼40,000–50,000 reads per cell. Demultiplexing, alignment to GRCh38 and UMI counting were performed using Cell Ranger v7.2.

##### Preprocessing and dimensionality reduction

Data were processed using Seurat v5.0 in R. Cells with fewer than 500 detected genes or >10% mitochondrial transcripts were excluded. Previously published single-cell RNA-seq data from one xenografted sample were incorporated into the analysis (GSE178912, GSM5400788).^24^ Processed data were retrieved from the Gene Expression Omnibus and processed alongside newly generated datasets using the same computational pipeline. Gene expression values were log-normalized and highly variable genes were identified using the variance stabilizing transformation method. Principal component analysis (PCA) was performed for dimensionality reduction, and low-dimensional visualization was generated using UMAP.

##### Clustering and definition of leukemic cell states

Initial exploratory clustering was performed using a shared nearest-neighbor graph and the Louvain algorithm. For the definition of leukemic transcriptional states, clustering was subsequently performed using hierarchical clustering (R function hclust) with Ward’s linkage (Ward.D2) applied to cosine distances. Cosine distance was computed after L2 normalization of the low-dimensional transcriptional space (PCA embeddings), such that Euclidean distance in this space is equivalent to cosine dissimilarity. The analysis was restricted to leukemic cells after exclusion of normal-like populations. To assess cluster robustness, silhouette widths were computed using the cluster R package based on the same distance matrix used for clustering. This analysis supported the stability of the identified leukemic states and guided the selection of the final clustering resolution.

##### Trajectory inference and gene program analysis

Trajectory inference and pseudotime ordering were performed to characterize transcriptional transitions across leukemic states. To position cells along the hematopoietic differentiation axis, enrichment scores for reference gene signatures (HSC-like, progenitor-like, GMP-like, pro-monocyte–like, myelocyte-like, and monocyte-like) were computed using Seurat gene set scoring. Cluster-associated gene programs were analyzed by pathway enrichment using clusterProfiler with Hallmark (MSigDB), KEGG, and Reactome gene sets. Significance was assessed using hypergeometric testing with Benjamini–Hochberg correction. Cell cycle activity was quantified using established G1/S and G2/M gene signatures.^49^ A proliferation score was computed as the average normalized expression of these signatures.

#### Targeted DNA sequencing and variant annotation

Genomic DNA was extracted using the Qiagen DNA/RNA Mini Kit and analyzed with the AmpliSeq Myeloid Panel (Illumina) sequenced on an Ion Torrent S5. Variants were called using Torrent Suite Variant Caller (TSVC v5.6). Annotation and filtering were performed with Ion Reporter v5.18 and complementary pipelines (Polydiag suite; OncoKB).^50^

#### DNA methylation profiling and analysis (infinium MethylationEPIC)

Genomic DNA (500 ng) was bisulfite-converted with the EZ DNA Methylation Kit and hybridized to Infinium MethylationEPIC BeadChips (Illumina). BeadChips were scanned on an iScan. IDAT files were imported into GenomeStudio for probe detection. Quality control, Noob normalization, and β- and M-value calculation were performed using SeSAMe (v1.14.2), including masking of probes affected by SNPs or potential copy-number loss. Differentially methylated positions were defined using an effect-size threshold (|Δβ| ≥ 0.15) and multiple-testing correction (FDR <0.05). Hierarchical clustering of differentially methylated positions was performed using nclust. Transcription factor (TF) target enrichment for methylation-associated genes was performed by over-representation testing, with FDR correction.

#### Copy number inference from methylation arrays

Copy number variations (CNVs) were inferred from Infinium MethylationEPIC array signal intensities. Total probe intensities (methylated + unmethylated) were extracted and normalized using the SeSAMe pipeline. Log2 signal ratios relative to matched baseline samples were computed and segmented using circular binary segmentation (CBS). Segment mean values (seg.mean) represent the average log2 copy number ratio across each genomic region. Significant CNV segments were defined based on segmentation *p*-values and effect size thresholds. Gains and losses were inferred from positive and negative segment means, respectively.

#### Genome stability metrics across serial passages

To summarize genomic divergence across serial passages, per-sample mutation profiles (variant allele frequencies; VAFs) were compared to matched *P*_*0*_ samples using distance metrics. Mutation divergence was quantified as Jensen–Shannon distance (JSD) between the VAF distributions of each sample and its matched *P*_*0*_ baseline. Copy-number variation (CNV) change was summarized as the sum of absolute segment mean values (Σ|seg.mean|) for differential segments (*P*_*1*_ vs. *P*_*0*_ and *P*_*2*_ vs. *P*_*0*_), with a value of 0 assigned to *P*_*0*_ samples and to PDXs with no detected differential CNV segments; CNV change was displayed as a square-root–scaled point size for visualization (genomics trajectory panel).

#### Bulk RNA sequencing

Total RNA (RIN >8) was poly(A)-selected (TruSeq Stranded mRNA Library Prep, Illumina) and sequenced on a NovaSeq 6000. Reads were aligned with STAR (v2.6.1day; hg38) and quantified with Salmon (v1.5.2). Differential expression analyses were performed using DESeq2 (v1.34.0) with batch correction where applicable using limma.

#### Proteomics (DIA-MS)

Cell pellets (∼1 × 10^7^ cells) were lysed in 1% SDS/50 mM TEAB supplemented with TCEP and chloroacetamide. Proteins (20 μg) were processed using the SP3-iST kit, including bead clean-up and overnight trypsin/LysC digestion. Peptides were separated by nanoLC (Vanquish *Neo* UHPLC; 135-min gradient) and analyzed on an Orbitrap Fusion Lumos operated in DIA mode. Raw files were processed in Spectronaut v19 using directDIA+ against combined human and mouse UniProt proteomes with 1% peptide/protein FDR. Protein group intensities were median-normalized and differential abundance was assessed using limma (|log2FC| ≥ 0.58; q ≤ 0.05).

#### Ribosome profiling (ribo-seq)

Cells (1 × 10^7^) were lysed in ice-cold polysome buffer containing cycloheximide and processed by sucrose gradient ultracentrifugation (10–50% sucrose). 80S monosome fractions were collected and digested with RNase I. Ribosome-protected fragments (25–34 nt) were size-selected, ligated, reverse-transcribed, circularized and PCR-indexed. Libraries were sequenced on a NovaSeq 6000 (1 × 100 bp). Quality control and periodicity metrics were assessed using RiboSeQC.

#### rRNA 2′-*O*-methylation profiling (RiboMeth-seq)

RiboMethSeq was performed as described previously^51^^,^^52^ using partial alkaline hydrolysis of total RNA followed by Illumina sequencing (1 × 75 bp). Reads were adapter-trimmed (Trimmomatic), aligned end-to-end to the human rRNA reference (NR_046235) using Bowtie, and 5′/3′ end counts per nucleotide were extracted using rRMSAnalyzer. C-scores were calculated in a ±6 nt window; variable 2′-*O*-methylated sites were defined as those with an interquartile range (IQR) of C-scores >0.15 across samples.

#### Ex-vivo culture and chemical screen

Drug sensitivity assays were conducted on bulk xenograft samples with high leukemic infiltration, in contrast to omics analyses performed on FACS-purified leukemic blasts. Samples were used directly when human leukemic infiltration exceeded 70%, or after murine cell depletion using anti-mouse CD45 magnetic separation when infiltration was lower. Cells were cultured in IMDM/BIT medium supplemented with recombinant human SCF, FLT3L, TPO, IL-3, IL-6 and G-CSF (50/50/25/10/10/10 ng/mL) and 50 μM β-mercaptoethanol, as reported.^53^ For screening, cells were seeded at 1 × 10^6^ cells/mL in 384-well plates and exposed for 48 h to 1 μM compounds from the MedChemExpress anti-cancer library (3,247 compounds). Viability was quantified using ATPlite luminescence on a SpectraMax L384 reader, which specifically reflects human AML cell viability.

#### Drug response metrics and statistical framework

For each compound and each PDX model, differential sensitivity across evolution was defined as Δ viability = viability(*P*_*2*_) − viability(*P*_*0*_). Analyses were restricted to seven matched PDX models with complete *P*_*0*_ and *P*_*2*_ data. Complete *P*_*1*_ data were available for TUH06, TUH10, TUH76, and TUH102 only and were included for visualization but were not used for primary statistical testing. For each compound, summary metrics across PDX models included the mean Δ, median Δ, 10% trimmed mean, interquartile range (IQR), median absolute deviation (MAD), and the number of models showing increased sensitivity (n_neg, defined as Δ < 0). Compound-level directionality was tested using a two-sided Wilcoxon signed-rank test against Δ = 0. Concordance across models was additionally assessed using a binomial sign test on n_neg (excluding Δ = 0 values). Compounds were categorized into robustness tiers based on directional concordance: High robustness was defined as 7/7 models with Δ < 0 and IQR ≤40; Moderate robustness as ≥6/7 models with Δ < 0 and IQR ≤40; Variable otherwise. These criteria were defined *a priori* to identify evolution-associated vulnerabilities with consistent cross-model effects. For visualization of prioritized compounds, *P*_*0*_ samples were displayed on the left and *P*_*2*_ samples on the right. To avoid artificial cross-passage clustering, hierarchical clustering of compounds was performed independently within *P*_*0*_ and *P*_*2*_ groups using distance = 1 − Spearman correlation and average linkage. *P*_*1*_ samples were omitted from clustering to maintain consistency with the primary statistical comparison (*P*_*2*_ vs. *P*_*0*_).

#### Multi-omics integration and trajectory analyses

To compare the timing and magnitude of molecular divergence across layers, sample-level distances to matched *P*_*0*_ baselines were computed for each omics layer and visualized as trajectories across passages (*P*_*0*_, *P*_*1*_, *P*_*2*_). For transcriptome and proteome, distances were defined as 1 − Spearman correlation to matched *P*_*0*_. For methylation, a corresponding distance metric (1 − Spearman) was computed on β-values across informative CpGs. A combined multi-omics embedding was generated by applying UMAP (uwot) to the matrix of per-sample omics distances to *P*_*0*_ (genomics, methylome, transcriptome, proteome), with trajectories plotted by connecting matched samples from the same PDX across passages. Pathway enrichment analyses were performed using fgsea for RNA-seq (ranked log2 fold changes) and over-representation analysis for proteomics using curated Hallmark and Reactome gene sets.

#### Multiple factor analysis (MFA)

An unsupervised Multiple Factor Analysis (MFA) was performed to identify latent axes of coordinated multi-omics variation across samples. Input blocks included genomics (mutation/CNV summary features), methylome (selected CpG features or module scores), transcriptome (gene-level log2FC or pathway scores), and proteome (protein-level log2FC or pathway scores), after aligning samples and scaling each block. MFA was computed using FactoMineR::MFA with quantitative variables (type = ‘s'). An integrated trajectory score was defined by projecting each sample onto the *P*_*0*_-to-*P*_*2*_ direction along the first MFA dimension (Dim1), and compared across passages.

#### Western blotting

Cell lysates were resolved on 4–12% Bis-Tris gels, transferred to 0.2 μm nitrocellulose membranes, and probed with antibodies against GSPT1, GSPT2, CRBN, XIAP, c-IAP1, or β-actin (key resources table). HRP-conjugated secondary antibodies were detected by chemiluminescence and imaged on a Syngene PXi.

### Quantification and statistical analysis

Statistical analyses were performed in R (v4.4.1). Unless stated otherwise, tests were two-sided and *p* values <0.05 were considered significant. For paired comparisons across serial passages within the same PDX model or patient, paired t-tests or Wilcoxon signed-rank tests were used depending on data distribution and sample size; for multi-group comparisons, one-way repeated-measures ANOVA with Tukey correction or the non-parametric Friedman test was used as appropriate. For multiple comparisons within a figure panel, *p* values were adjusted using Holm correction unless stated otherwise.

## Supplemental information

Document S1. Figures S1–S6 and Table S17

Table S1. Flow cytometry quantification of leukemic cell populations during serial xenotransplantation, related to Figure 1

Table S2. Flow cytometry quantification of candidate LSC phenotypes across serial xenotransplantation, related to Figure 1

Table S3. Low-input leukemia-initiating cell assay raw data across serial xenograft passages, related to Figure 1

Table S4. Limiting dilution analysis raw data across serial xenograft passages, related to Figure 1

Table S5. Cluster-associated marker genes from single-cell RNA-seq, related to Figure 2

Table S6. Functional enrichment of leukemic single-cell cluster-associated programs, related to Figure 2

Table S7. Variant allele frequencies of somatic mutations detected across PDX models and serial propagation stages, related to Figure 3

Table S8. Multi-omics divergence metrics across PDX propagation, related to Figure 3

Table S9. Literature-derived LSC and hematopoietic progenitor gene signatures used for enrichment analyses, related to Figure 3

Table S10. Propagation score validation in independent AML patient cohorts, related to Figure 3

Table S11. Multiple factor analysis coordinates and integrated progression scores, related to Figure 4

Table S12. Reactome pathway enrichment analysis of transcriptomic and proteomic changes during leukemic propagation, related to Figure 4

Table S13. Ribo-Seq translation efficiency analysis across xenograft passages, related to Figure 4

Table S14. RiboMethSeq rRNA 2′-O-methylation scores across AML samples, related to Figure 4

Table S15. Compound-level summary of pharmacological screening responses across serial PDX stages, related to Figure 5

Table S16. Raw pharmacological screening viability data across AML PDX models and passages, related to Figure 5

Document S2. Article plus supplemental information

## References

[1] Döhner H., Weisdorf D.J., Bloomfield C.D. (2015). Acute Myeloid Leukemia. N. Engl. J. Med..

[2] Döhner H., Estey E., Grimwade D., Amadori S., Appelbaum F.R., Büchner T., Dombret H., Ebert B.L., Fenaux P., Larson R.A. (2017). Diagnosis and management of AML in adults: 2017 ELN recommendations from an international expert panel. Blood.

[3] Dick J.E. (2008). Stem cell concepts renew cancer research. Blood.

[4] Bonnet D., Dick J.E. (1997). Human acute myeloid leukemia is organized as a hierarchy that originates from a primitive hematopoietic cell. Nat. Med..

[5] Dalerba P., Cho R.W., Clarke M.F. (2007). Cancer stem cells: models and concepts. Annu. Rev. Med..

[6] Kreso A., Dick J.E. (2014). Evolution of the cancer stem cell model. Cell Stem Cell.

[7] Ishikawa F., Yoshida S., Saito Y., Hijikata A., Kitamura H., Tanaka S., Nakamura R., Tanaka T., Tomiyama H., Saito N. (2007). Chemotherapy-resistant human AML stem cells home to and engraft within the bone-marrow endosteal region. Nat. Biotechnol..

[8] Sarry J.-E., Murphy K., Perry R., Sanchez P.V., Secreto A., Keefer C., Swider C.R., Strzelecki A.-C., Cavelier C., Récher C. (2011). Human acute myelogenous leukemia stem cells are rare and heterogeneous when assayed in NOD/SCID/IL2Rγc-deficient mice. J. Clin. Investig..

[9] Jones C.L., Stevens B.M., D’Alessandro A., Reisz J.A., Culp-Hill R., Nemkov T., Pei S., Khan N., Adane B., Ye H. (2018). Inhibition of Amino Acid Metabolism Selectively Targets Human Leukemia Stem Cells. Cancer Cell.

[10] Lagadinou E.D., Sach A., Callahan K., Rossi R.M., Neering S.J., Minhajuddin M., Ashton J.M., Pei S., Grose V., O’Dwyer K.M. (2013). BCL-2 inhibition targets oxidative phosphorylation and selectively eradicates quiescent human leukemia stem cells. Cell Stem Cell.

[11] Pollyea D.A., Stevens B.M., Jones C.L., Winters A., Pei S., Minhajuddin M., D’Alessandro A., Culp-Hill R., Riemondy K.A., Gillen A.E. (2018). Venetoclax with azacitidine disrupts energy metabolism and targets leukemia stem cells in patients with acute myeloid leukemia. Nat. Med..

[12] van Galen P., Hovestadt V., Wadsworth Ii M.H., Hughes T.K., Griffin G.K., Battaglia S., Verga J.A., Stephansky J., Pastika T.J., Lombardi Story J. (2019). Single-Cell RNA-Seq Reveals AML Hierarchies Relevant to Disease Progression and Immunity. Cell.

[13] Goardon N., Marchi E., Atzberger A., Quek L., Schuh A., Soneji S., Woll P., Mead A., Alford K.A., Rout R. (2011). Coexistence of LMPP-like and GMP-like leukemia stem cells in acute myeloid leukemia. Cancer Cell.

[14] Boyd A.L., Lu J., Hollands C.G., Alsostovar L., Murali S., Reid J.C., Ye W., Vandersluis S., Johnson P., ElRafie A. (2023). Leukemic progenitor compartment serves as a prognostic measure of cancer stemness in patients with acute myeloid leukemia. Cell Rep. Med..

[15] Shlush L.I., Mitchell A., Heisler L., Abelson S., Ng S.W.K., Trotman-Grant A., Medeiros J.J.F., Rao-Bhatia A., Jaciw-Zurakowsky I., Marke R. (2017). Tracing the origins of relapse in acute myeloid leukaemia to stem cells. Nature.

[16] Matyskiela M.E., Lu G., Ito T., Pagarigan B., Lu C.-C., Miller K., Fang W., Wang N.-Y., Nguyen D., Houston J. (2016). A novel cereblon modulator recruits GSPT1 to the CRL4(CRBN) ubiquitin ligase. Nature.

[17] Hennessy E.J., Adam A., Aquila B.M., Castriotta L.M., Cook D., Hattersley M., Hird A.W., Huntington C., Kamhi V.M., Laing N.M. (2013). Discovery of a novel class of dimeric Smac mimetics as potent IAP antagonists resulting in a clinical candidate for the treatment of cancer (AZD5582). J. Med. Chem..

[18] Taussig D.C., Vargaftig J., Miraki-Moud F., Griessinger E., Sharrock K., Luke T., Lillington D., Oakervee H., Cavenagh J., Agrawal S.G. (2010). Leukemia-initiating cells from some acute myeloid leukemia patients with mutated nucleophosmin reside in the CD34(-) fraction. Blood.

[19] Hassan N., Yang J., Wang J.Y. (2020). An Improved Protocol for Establishment of AML Patient-Derived Xenograft Models. STAR Protoc..

[20] Pei S., Pollyea D.A., Gustafson A., Stevens B.M., Minhajuddin M., Fu R., Riemondy K.A., Gillen A.E., Sheridan R.M., Kim J. (2020). Monocytic Subclones Confer Resistance to Venetoclax-Based Therapy in Patients with Acute Myeloid Leukemia. Cancer Discov..

[21] Ho J.M., Dobson S.M., Voisin V., McLeod J., Kennedy J.A., Mitchell A., Jin L., Eppert K., Bader G., Minden M.D. (2020). CD200 expression marks leukemia stem cells in human AML. Blood Adv..

[22] Boyd A.L., Aslostovar L., Reid J., Ye W., Tanasijevic B., Porras D.P., Shapovalova Z., Almakadi M., Foley R., Leber B. (2018). Identification of Chemotherapy-Induced Leukemic-Regenerating Cells Reveals a Transient Vulnerability of Human AML Recurrence. Cancer Cell.

[23] Hu Y., Smyth G.K. (2009). ELDA: extreme limiting dilution analysis for comparing depleted and enriched populations in stem cell and other assays. J. Immunol. Methods.

[24] Bosc C., Saland E., Bousard A., Gadaud N., Sabatier M., Cognet G., Farge T., Boet E., Gotanègre M., Aroua N. (2021). Mitochondrial inhibitors circumvent adaptive resistance to venetoclax and cytarabine combination therapy in acute myeloid leukemia. Nat. Cancer.

[25] Zeng A.G.X., Bansal S., Jin L., Mitchell A., Chen W.C., Abbas H.A., Chan-Seng-Yue M., Voisin V., van Galen P., Tierens A. (2022). A cellular hierarchy framework for understanding heterogeneity and predicting drug response in acute myeloid leukemia. Nat. Med..

[26] Ng S.W.K., Mitchell A., Kennedy J.A., Chen W.C., McLeod J., Ibrahimova N., Arruda A., Popescu A., Gupta V., Schimmer A.D. (2016). A 17-gene stemness score for rapid determination of risk in acute leukaemia. Nature.

[27] Kawashima N., Ishikawa Y., Kim J.H., Ushijima Y., Akashi A., Yamaguchi Y., Hattori H., Nakashima M., Ikeno S., Kihara R. (2022). Comparison of clonal architecture between primary and immunodeficient mouse-engrafted acute myeloid leukemia cells. Nat. Commun..

[28] Hackl H., Steinleitner K., Lind K., Hofer S., Tosic N., Pavlovic S., Suvajdzic N., Sill H., Wieser R. (2015). A gene expression profile associated with relapse of cytogenetically normal acute myeloid leukemia is enriched for leukemia stem cell genes. Leuk. Lymphoma.

[29] Li S., Garrett-Bakelman F.E., Chung S.S., Sanders M.A., Hricik T., Rapaport F., Patel J., Dillon R., Vijay P., Brown A.L. (2016). Distinct evolution and dynamics of epigenetic and genetic heterogeneity in acute myeloid leukemia. Nat. Med..

[30] Giacopelli B., Wang M., Cleary A., Wu Y.-Z., Schultz A.R., Schmutz M., Blachly J.S., Eisfeld A.-K., Mundy-Bosse B., Vosberg S. (2021). DNA methylation epitypes highlight underlying developmental and disease pathways in acute myeloid leukemia. Genome Res..

[31] Ingolia N.T., Ghaemmaghami S., Newman J.R.S., Weissman J.S. (2009). Genome-Wide Analysis in Vivo of Translation with Nucleotide Resolution Using Ribosome Profiling. Science.

[32] Zhou F., Aroua N., Liu Y., Rohde C., Cheng J., Wirth A.-K., Fijalkowska D., Göllner S., Lotze M., Yun H. (2023). A Dynamic rRNA Ribomethylome Drives Stemness in Acute Myeloid Leukemia. Cancer Discov..

[33] Zhao Y., Rai J., Cohen L.N., Shu Y., Xu C., Goswami H., Chen X., Jin H., Marchand V., Motorin Y. (2026). 2′-O-Methylation maintains ribosome structural and translation integrity. Mol. Cell.

[34] Lin S., Larrue C., Scheidegger N.K., Seong B.K.A., Dharia N.V., Kuljanin M., Wechsler C.S., Kugener G., Robichaud A.L., Conway A.S. (2022). An In Vivo CRISPR Screening Platform for Prioritizing Therapeutic Targets in AML. Cancer Discov..

[35] Sellar R.S., Sperling A.S., Słabicki M., Gasser J.A., McConkey M.E., Donovan K.A., Mageed N., Adams D.N., Zou C., Miller P.G. (2022). Degradation of GSPT1 causes TP53-independent cell death in leukemia while sparing normal hematopoietic stem cells. J. Clin. Investig..

[36] Farge T., Saland E., de Toni F., Aroua N., Hosseini M., Perry R., Bosc C., Sugita M., Stuani L., Fraisse M. (2017). Chemotherapy-Resistant Human Acute Myeloid Leukemia Cells Are Not Enriched for Leukemic Stem Cells but Require Oxidative Metabolism. Cancer Discov..

[37] Ho T.-C., LaMere M., Stevens B.M., Ashton J.M., Myers J.R., O’Dwyer K.M., Liesveld J.L., Mendler J.H., Guzman M., Morrissette J.D. (2016). Evolution of acute myelogenous leukemia stem cell properties after treatment and progression. Blood.

[38] Haubner S., Perna F., Köhnke T., Schmidt C., Berman S., Augsberger C., Schnorfeil F.M., Krupka C., Lichtenegger F.S., Liu X. (2019). Coexpression profile of leukemic stem cell markers for combinatorial targeted therapy in AML. Leukemia.

[39] Zeijlemaker W., Kelder A., Oussoren-Brockhoff Y.J.M., Scholten W.J., Snel A.N., Veldhuizen D., Cloos J., Ossenkoppele G.J., Schuurhuis G.J. (2016). A simple one-tube assay for immunophenotypical quantification of leukemic stem cells in acute myeloid leukemia. Leukemia.

[40] van Rhenen A., van Dongen G.A.M.S., Kelder A., Rombouts E.J., Feller N., Moshaver B., Stigter-van Walsum M., Zweegman S., Ossenkoppele G.J., Jan Schuurhuis G. (2007). The novel AML stem cell associated antigen CLL-1 aids in discrimination between normal and leukemic stem cells. Blood.

[41] Hansen J.L., Moore P.B., Steitz T.A. (2003). Structures of five antibiotics bound at the peptidyl transferase center of the large ribosomal subunit. J. Mol. Biol..

[42] Hashimoto M., Saito Y., Nakagawa R., Ogahara I., Takagi S., Takata S., Amitani H., Endo M., Yuki H., Ramilowski J.A. (2021). Combined inhibition of XIAP and BCL2 drives maximal therapeutic efficacy in genetically diverse aggressive acute myeloid leukemia. Nat. Cancer.

[43] Surka C., Jin L., Mbong N., Lu C.-C., Jang I.S., Rychak E., Mendy D., Clayton T., Tindall E., Hsu C. (2021). CC-90009, a novel cereblon E3 ligase modulator, targets acute myeloid leukemia blasts and leukemia stem cells. Blood.

[44] Döhner H., Wei A.H., Appelbaum F.R., Craddock C., DiNardo C.D., Dombret H., Ebert B.L., Fenaux P., Godley L.A., Hasserjian R.P. (2022). Diagnosis and management of AML in adults: 2022 recommendations from an international expert panel on behalf of the ELN. Blood.

[45] Kilham H. (2018). Re: Sexual Harassment. J. Paediatr. Child Health.

[46] Ahmed R., Botezatu B., Nanthakumar M., Kaloti T., Harky A. (2021). Surgery for heart failure: Treatment options and implications. J. Card. Surg..

[47] Bolger A.M., Lohse M., Usadel B. (2014). Trimmomatic: a flexible trimmer for Illumina sequence data. Bioinformatics.

[48] Sabatier M., Birsen R., Lauture L., Mouche S., Angelino P., Dehairs J., Goupille L., Boussaid I., Heiblig M., Boet E. (2023). C/EBPα Confers Dependence to Fatty Acid Anabolic Pathways and Vulnerability to Lipid Oxidative Stress-Induced Ferroptosis in FLT3-Mutant Leukemia. Cancer Discov..

[49] Tirosh I., Izar B., Prakadan S.M., Wadsworth M.H., Treacy D., Trombetta J.J., Rotem A., Rodman C., Lian C., Murphy G. (2016). Dissecting the multicellular ecosystem of metastatic melanoma by single-cell RNA-seq. Science.

[50] Decroocq J., Birsen R., Montersino C., Chaskar P., Mano J., Poulain L., Friedrich C., Alary A.-S., Guermouche H., Sahal A. (2022). RAS activation induces synthetic lethality of MEK inhibition with mitochondrial oxidative metabolism in acute myeloid leukemia. Leukemia.

[51] Marcel V., Kielbassa J., Marchand V., Natchiar K.S., Paraqindes H., Nguyen Van Long F., Ayadi L., Bourguignon-Igel V., Lo Monaco P., Monchiet D. (2020). Ribosomal RNA 2’O-methylation as a novel layer of inter-tumour heterogeneity in breast cancer. NAR Cancer.

[52] Monaco P.L., Marcel V., Diaz J.-J., Catez F. (2018). 2’-O-Methylation of Ribosomal RNA: Towards an Epitranscriptomic Control of Translation?. Biomolecules.

[53] Larrue C., Mouche S., Angelino P., Sajot M., Birsen R., Kosmider O., Mckee T., Vergez F., Recher C., Mas V.M.-D. (2024). Targeting ferritinophagy impairs quiescent cancer stem cells in acute myeloid leukemia in vitro and in vivo models. Sci. Transl. Med..

