# Supplementary material for: Non-genetic remodeling drives leukemia propagation and reveals actionable vulnerabilities in acute myeloid leukemia
Source: Cell Rep Med. 2026 Aug 31;7(9):103017. doi: 10.1016/j.xcrm.2026.103017 (PMC13589494; doi:10.1016/j.xcrm.2026.103017)
Supplement: Supplementary file 1 [file mmc1.pdf]

Cell Reports Medicine, Volume *7*

Supplemental information

Non-genetic remodeling drives leukemia propagation and reveals actionable vulnerabilities in acute myeloid leukemia

Clement Larrue, Paolo Angelino, Sarah Mouche, Alexa S. Green, Emeline Boet, Ismael Boussaid, Claudia Perez-Carretero, Rudy Birsen, Maxime Sajot, Thomas A. Mckee, Ambrine Sahal, Nicolas Chapuis, François Vergez, Christian Recher, Véronique Mansat-De Mas, Olivier Kosmider, Pierre Sujobert, Frederic Catez, Jean-Jacques Diaz, Jean-Emmanuel Sarry, Petros Tsantoulis, and Jerome Tamburini

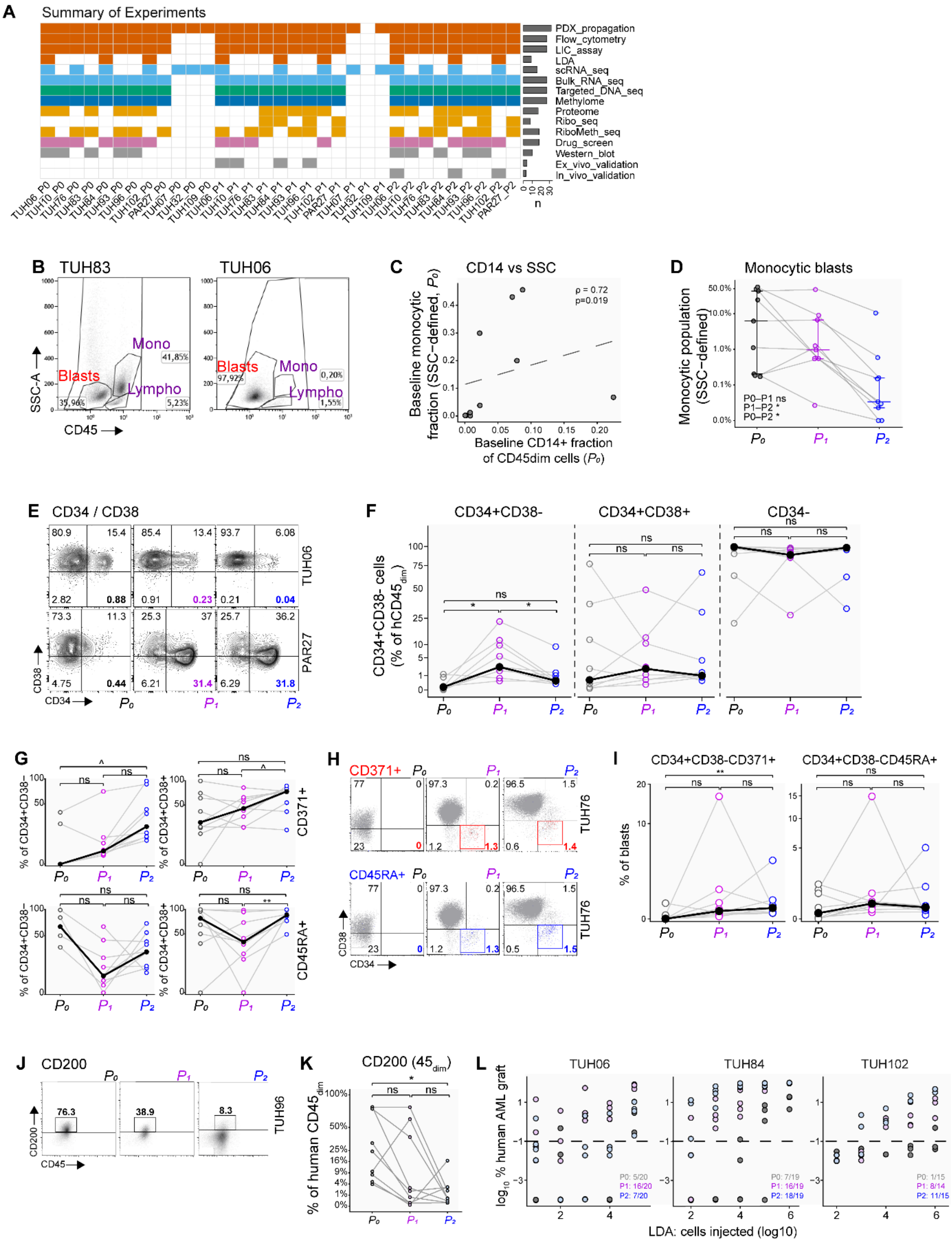

Figure S1. Serial xenotransplantation enriches leukemia-initiating cell capacities in immunodeficient mice. Related to Figure 1. A. Heatmap summarizing the experimental assays performed for each AML PDX sample

across serial propagation stages (P_0–P_2). Columns represent individual samples and rows indicate assays. Colored cells denote assays performed, grouped by experimental category, while white cells indicate assays not performed. The bar plot indicates the number of samples analyzed per assay (n), illustrating dataset attrition across experimental layers. **B.** Representative flow cytometry plot (TUH83 and TUH06, P_0) showing gating strategy for CD45 versus side scatter (SSC-A), identifying $CD45_{dim}$ blasts, SSC-defined monocytic ("Mono") population, and lymphocytes ("Lympho"). **C.** Correlation between baseline $CD14^+$ fraction within $CD45_{dim}$ cells and SSC-defined monocytic fraction at P_0. Spearman correlation coefficient (ρ) and p-value are indicated. **D.** Longitudinal quantification of the SSC-defined monocytic population across P_0, P_1, and P_2. Lines connect matched models; statistical comparisons between stages are shown. **E-F.** CD34 versus CD38 gating and quantification of blast immunophenotypic subsets across passages. **E.** Representative flow cytometry plots illustrating CD34/CD38 distribution at P_0, P_1, and P_2. **F.** Quantification of $CD34^+CD38^-$, $CD34^+CD38^+$, and $CD34^-$ fractions among human $CD45_{dim}$ blasts across serial propagation. **G.** Expression of LSC-associated markers across immunophenotypic blast subsets. CD371 and CD45RA expression is quantified within $CD34^+CD38^-$ and $CD34^+CD38^+$ blast populations across passages. **H-I.** Longitudinal quantification of specific LSC-like subpopulations including $CD34^+CD38^-CD371^+$ and $CD34^+CD38^-CD45RA^+$ cells among total blasts across P_0–P_2. **H.** Representative panels. **I.** Quantifications with each line representing a matched PDX model. **J-K.** CD200 expression within human $CD45_{dim}$ blasts and quantification across passages. **J.** Representative flow cytometry plots showing CD200 expression within human CD45dim cells across passages. **K.** Quantification of $CD200^+$ cells within the CD45dim blast compartment across serial propagation. **L.** Limiting dilution assays (LDA) across P_0, P_1, and P_2 in TUH06, TUH84, and TUH102. The proportion of engrafted mice at each cell dose is indicated (number of engrafted mice/total). Y-axis shows log10 human AML graft (%). All points represent individual mice; lines connect matched serial samples.

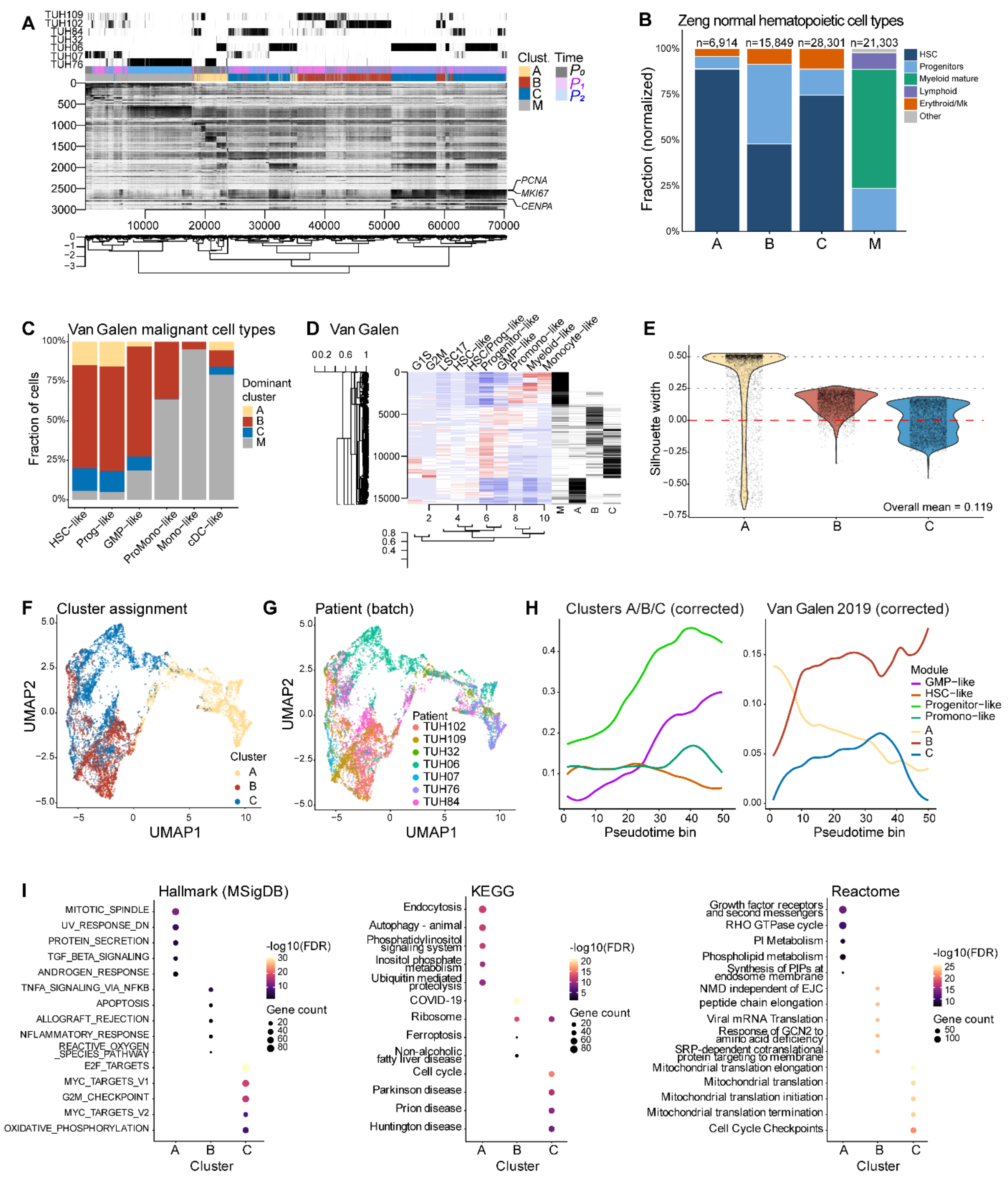

Figure S2. Leukemic propagation shifts the balance of conserved cell states. Related to Figure 2. A. Heatmap of gene expression across leukemic single cells from all AML samples analyzed by scRNA-seq. Cells are ordered by hierarchical clustering, with annotation tracks indicating patient identity and xenograft stage. Major transcriptional clusters (A–C and M) are indicated. Selected marker genes associated with proliferative programs (e.g., *PCNA*, *MKI67*) are shown. **B.** Projection of leukemic clusters (A–C and M) onto a normal hematopoietic reference dataset

(Zeng et al.). Stacked bar plots show the normalized fraction of cells assigned to each reference cell type within each cluster. **C.** Projection of leukemic clusters (A/B/C/M) onto an independent AML reference dataset (van Galen et al.). Bar plots show the fraction of cells assigned to each malignant cell type. **D.** Heatmap showing the distribution of reference transcriptional programs from van Galen et al. across leukemic single cells ordered by hierarchical clustering. Annotation tracks indicate cluster assignment (A–C and M). **E.** Silhouette analysis assessing clustering robustness across leukemic single cells. Violin plots show silhouette width distributions for clusters A–C. Positive values indicate consistent cluster assignment, whereas values close to zero reflect partial overlap between neighboring transcriptional states. **F.** UMAP representation of leukemic single cells colored by cluster assignment. **G.** UMAP projection colored by patient identity. **H.** Pseudotime analysis across clusters A–C. Left, dynamic changes in corrected cluster contributions along pseudotime bins. Right, dynamic changes in representative van Galen-derived programs (HSPC-like, progenitor-like, and promonocyte-like) along the inferred trajectory. **I.** Functional enrichment analysis of gene programs associated with clusters A–C using Hallmark (MSigDB), KEGG, and Reactome gene sets. Each dot represents an enriched pathway; dot size reflects the number of contributing genes and color indicates the false discovery rate (−log10 FDR).

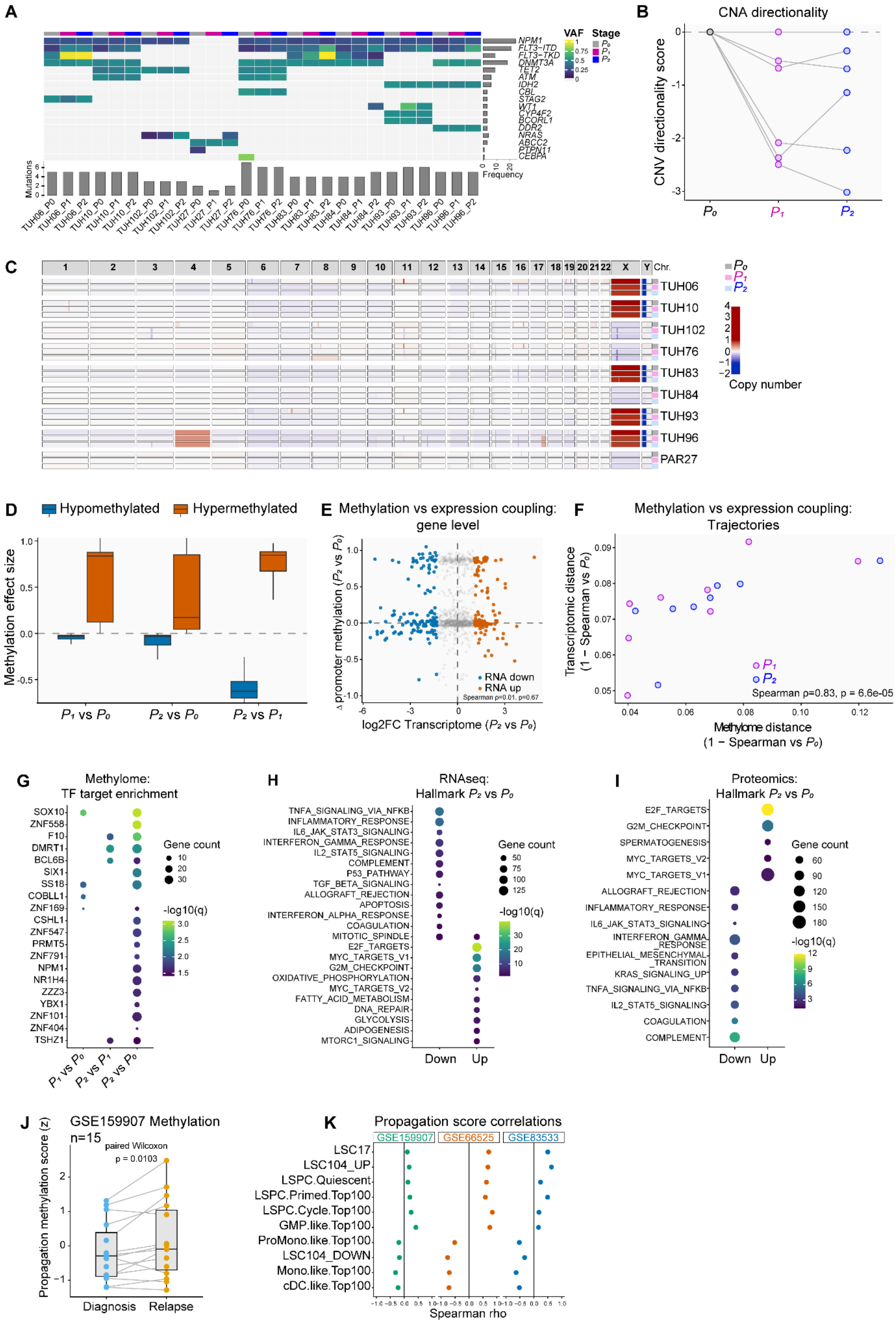
A
NPM1
FLT3-ITD
FLT3-TKD
DNMT3A
TET2
ATM
IDH2
CBL
STAG2
WT1
CYP4F2
BCORL1
DDR2
NRAS
ABCC2
PTPN11
CEBPA
VAF
Stage
Frequency
Mutations
B
CNA directionality
CNV directionality score
C
Chr.
TUH06
TUH10
TUH102
TUH76
TUH83
TUH84
TUH93
TUH96
PAR27
Copy number
D
Hypomethylated
Hypermethylated
Methylation effect size
E
Methylation vs expression coupling: gene level
Δ promoter methylation
RNA down
RNA up
Spearman ρ=0.01, p=0.67
log2FC Transcriptome
F
Methylation vs expression coupling: Trajectories
Transcriptomic distance
(1 − Spearman vs P₀)
Spearman ρ=0.83, p = 6.6e-05
Methylome distance
(1 − Spearman vs P₀)
G
Methylome:
TF target enrichment
SOX10
ZNF558
F10
DMRT1
BCL6B
SIX1
SS18
COBLL1
ZNF169
CSHL1
ZNF547
PRMT5
ZNF791
NPM1
NR1H4
ZZZ3
YBX1
ZNF101
ZNF404
TSHZ1
Gene count
-log10(q)
H
RNAseq:
Hallmark P₂ vs P₀
TNFA_SIGNALING_VIA_NFKB
INFLAMMATORY_RESPONSE
IL6_JAK_STAT3_SIGNALING
INTERFERON_GAMMA_RESPONSE
IL2_STAT5_SIGNALING
COMPLEMENT
P53_PATHWAY
TGF_BETA_SIGNALING
ALLOGRAFT_REJECTION
APOPTOSIS
INTERFERON_ALPHA_RESPONSE
COAGULATION
MITOTIC_SPINDLE
E2F_TARGETS
MYC_TARGETS_V1
G2M_CHECKPOINT
OXIDATIVE_PHOSPHORYLATION
MYC_TARGETS_V2
FATTY_ACID_METABOLISM
DNA_REPAIR
GLYCOLYSIS
ADIPOGENESIS
MTORC1_SIGNALING
Down
Up
Gene count
-log10(q)
I
Proteomics:
Hallmark P₂ vs P₀
E2F_TARGETS
G2M_CHECKPOINT
SPERMATOGENESIS
MYC_TARGETS_V2
MYC_TARGETS_V1
ALLOGRAFT_REJECTION
INFLAMMATORY_RESPONSE
IL6_JAK_STAT3_SIGNALING
INTERFERON_GAMMA_RESPONSE
EPITHELIAL_MESENCHYMAL_TRANSITION
KRAS_SIGNALING_UP
TNFA_SIGNALING_VIA_NFKB
IL2_STAT5_SIGNALING
COAGULATION
COMPLEMENT
Down
Up
Gene count
-log10(q)
J
GSE159907 Methylation
n=15
paired Wilcoxon
p = 0.0103
Propagation methylation score (z)
Diagnosis
Relapse
K
Propagation score correlations
GSE159907
GSE66525
GSE83533
LSC17
LSC104_UP
LSPC.Quiescent
LSPC.Primed.Top100
LSPC.Cycle.Top100
GMP.like.Top100
ProMono.like.Top100
LSC104_DOWN
Mono.like.Top100
cDC.like.Top100
Spearman rho

Figure S3. Progressive non-genetic remodeling is a major driver of leukemic evolution during serial xenotransplantation. Related to Figure 3. A. Variant allele frequencies (VAF) of recurrent mutations detected by targeted DNA sequencing in AML patient-derived xenografts across passages P_0, P_1, and P_2. Each column represents a sample and each row a gene. Color intensity reflects VAF, while grey indicates absence of mutation. The top annotation indicates xenograft stage. Bar plots show the number of mutated genes per sample (bottom) and mutation frequency across samples (right). **B.** Copy number alteration (CNA) directionality score across passages. Negative values indicate net loss; positive values indicate net gain relative to P_0. **C.** Genome-wide CNA profiles across chromosomes (Chr 1–22, X, Y) for each model and stage. **D.** Distribution of methylation effect sizes (Δβ). Hypermethylated and hypomethylated promoters are distinguished. **E.** Gene-level coupling between promoter methylation changes (Δ promoter methylation) and transcriptomic log2 fold change (P_2 vs P_0). Spearman correlation coefficient is indicated. **F.** Sample-level coupling between methylome and transcriptome distances relative to P_0 (1 − Spearman correlation across CpG sites and genes, respectively). Spearman ρ and p-value are shown. **G.** Transcription factor (TF) target enrichment analysis of differentially methylated promoters. Dot size indicates gene count; color encodes −log10(q-value). **H.** Hallmark pathway enrichment (RNA-seq; P_2 vs P_0). Dot size indicates gene set size; color encodes −log10(q-value). **I.** Hallmark pathway enrichment (proteomics; P_2 vs P_0). Dot size indicates gene set size; color encodes −log10(q-value). **J.** Propagation methylation scores were calculated from CpG sites associated with the propagation program and applied to paired AML samples from the GSE159907 cohort (n = 15 diagnosis–relapse pairs). Each dot represents one patient sample; lines connect matched diagnosis and relapse samples. Boxplots summarize the distribution across samples. **K.** Spearman correlations (ρ) between the PDX-derived propagation score and hematopoietic transcriptional signatures were computed independently in three AML patient datasets (GSE159907, GSE66525, GSE83533). Each point represents the correlation coefficient for one signature within a cohort.

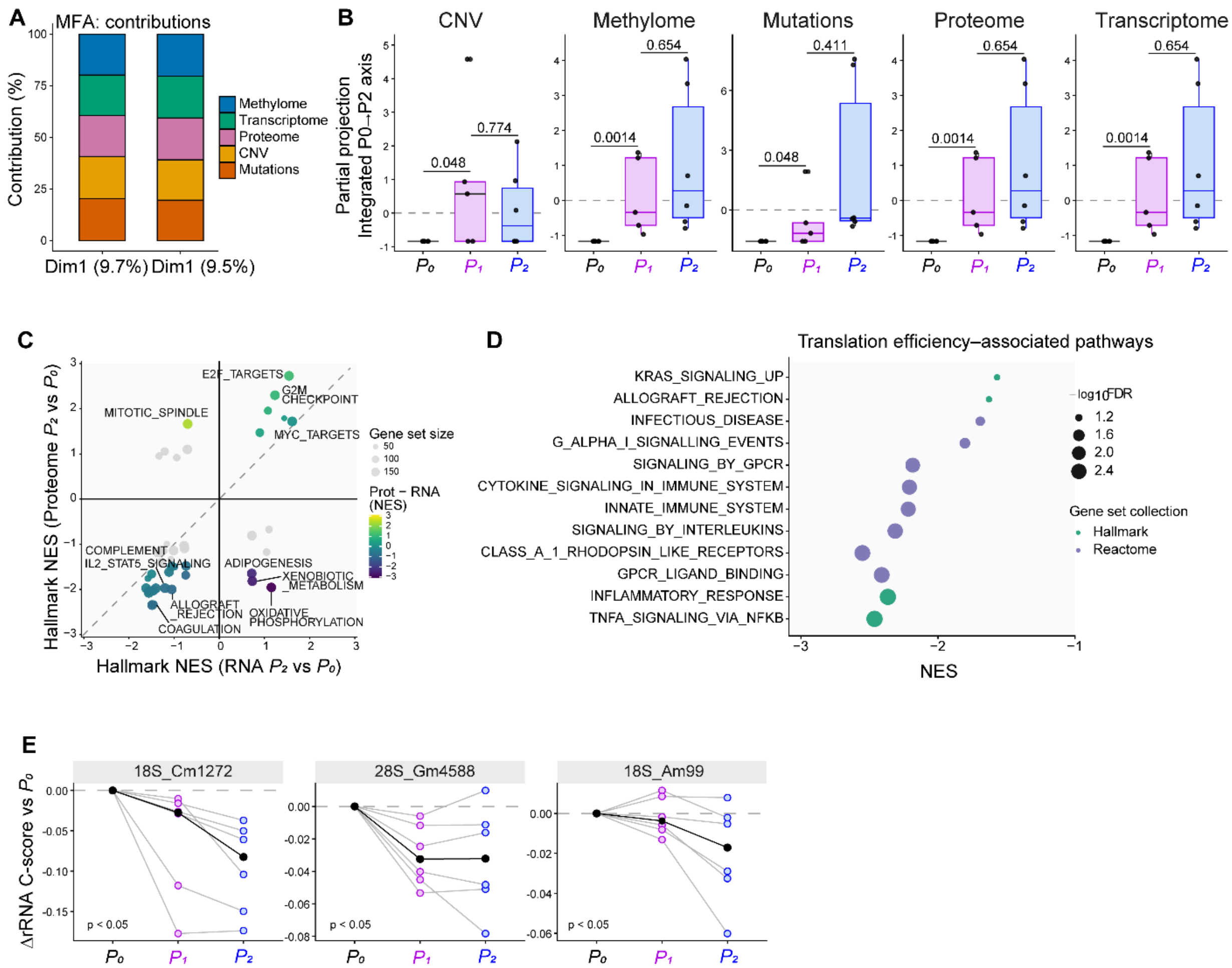

Figure S4. Integrated multi-omics progression uncovers translational activation and ribosome remodeling during leukemic adaptation. Related to Figure 4. A. Contribution of each molecular layer (methylome, transcriptome, proteome, CNV, mutations) to the primary MFA axis (Dim1). **B.** Partial projection of each omics layer onto the integrated $P_0 \rightarrow P_2$ axis, quantifying layer-specific displacement. **C.** Correlation between Hallmark pathway enrichment scores derived from RNA-seq and proteomics (P_2 vs P_0). Dot size represents gene set size; color encodes NES difference (protein − RNA). **D.** Enrichment of translation efficiency–associated pathways (Hallmark and Reactome). Dot size indicates gene count; color encodes −log10(FDR). **E.** Stage-dependent changes in rRNA 2′-O-methylation (Δ C-score vs P_0) for selected sites (18S_Cm1272, 28S_Gm4588, 18S_Am99). Lines connect matched serial samples; p-values indicated.

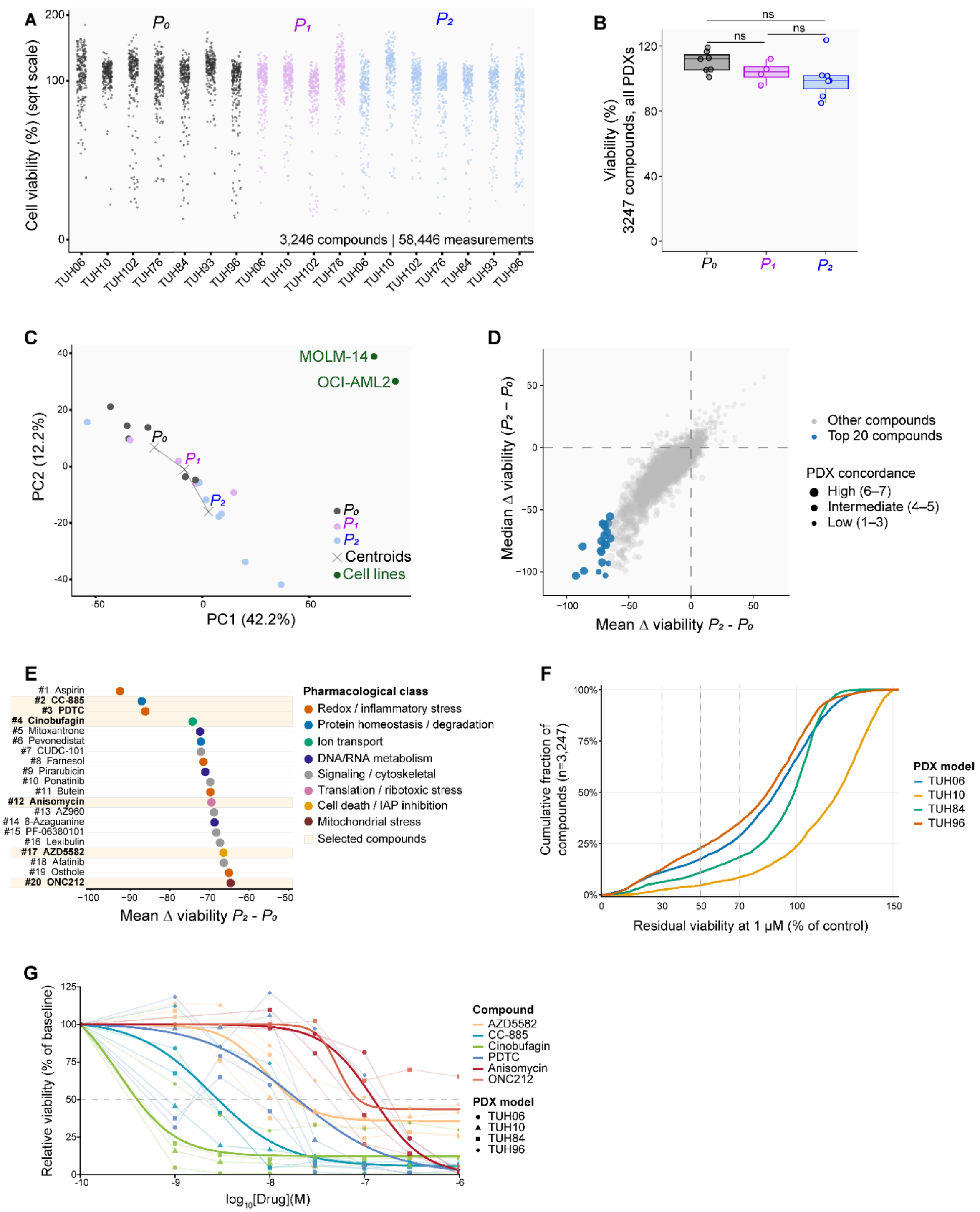

Figure S5. Stage-dependent reorganization of drug response profiles reveals a restricted set of robust vulnerabilities in advanced xenografts. Related to Figure 5. A. Distribution of normalized cell viability (%) across 3,247 compounds and 58,446 measurements in P_0, P_1, and P_2 samples. Viability values are normalized to vehicle-treated controls within each sample and displayed on a square-root scale. **B.** Summary distribution of viability across passages (all compounds pooled). **C.** PCA was performed on compound-level median viability

values across the shared compound library (n = 3,247 compounds). For each sample (PDX model or AML cell line), median viability per compound was calculated and standardized (z-score across samples) prior to PCA. Each point represents one biological sample. Stage centroids (P_0, P_1, P_2) are indicated. AML cell lines (OCI-AML2 and MOLM-14), screened under identical conditions, are shown for comparison. **D.** Differential response plot showing mean versus median Δ viability ($P_2 - P_0$) for all compounds. Top candidates are highlighted. PDX concordance categories indicate the number of PDX models showing enhanced activity at P_2 (High: 6–7 models; Intermediate: 4–5; Low: 1–3). IQR represents interquartile range of Δ viability across models. **E.** Top-20 compounds ranked by mean Δ viability (P_2–P_0) across matched xenograft models. Colors denote broad pharmacological classes, and shaded rows indicate compounds selected for in vivo evaluation. **F.** Cumulative distribution of residual viability values across the full 3,247-compound library tested at 1 μM in four P_2-stage xenografts used for dose-response assays. Curves show the fraction of compounds yielding residual viability at or below each indicated value. **G.** Cell viability was measured after 48 h of drug exposure and normalized to the lowest tested concentration within each compound–PDX trajectory. Symbols and faint lines indicate individual PDX models, whereas thick lines represent baseline-anchored logistic fits of the mean response across models at each concentration. AZD5582, CC-885, cinobufagin, and PDTC were evaluated in four independent P_2 PDX models (TUH06, TUH10, TUH84, and TUH96); anisomycin and ONC212 were evaluated in two models (TUH06 and TUH84). The dashed horizontal line denotes 50% relative viability.

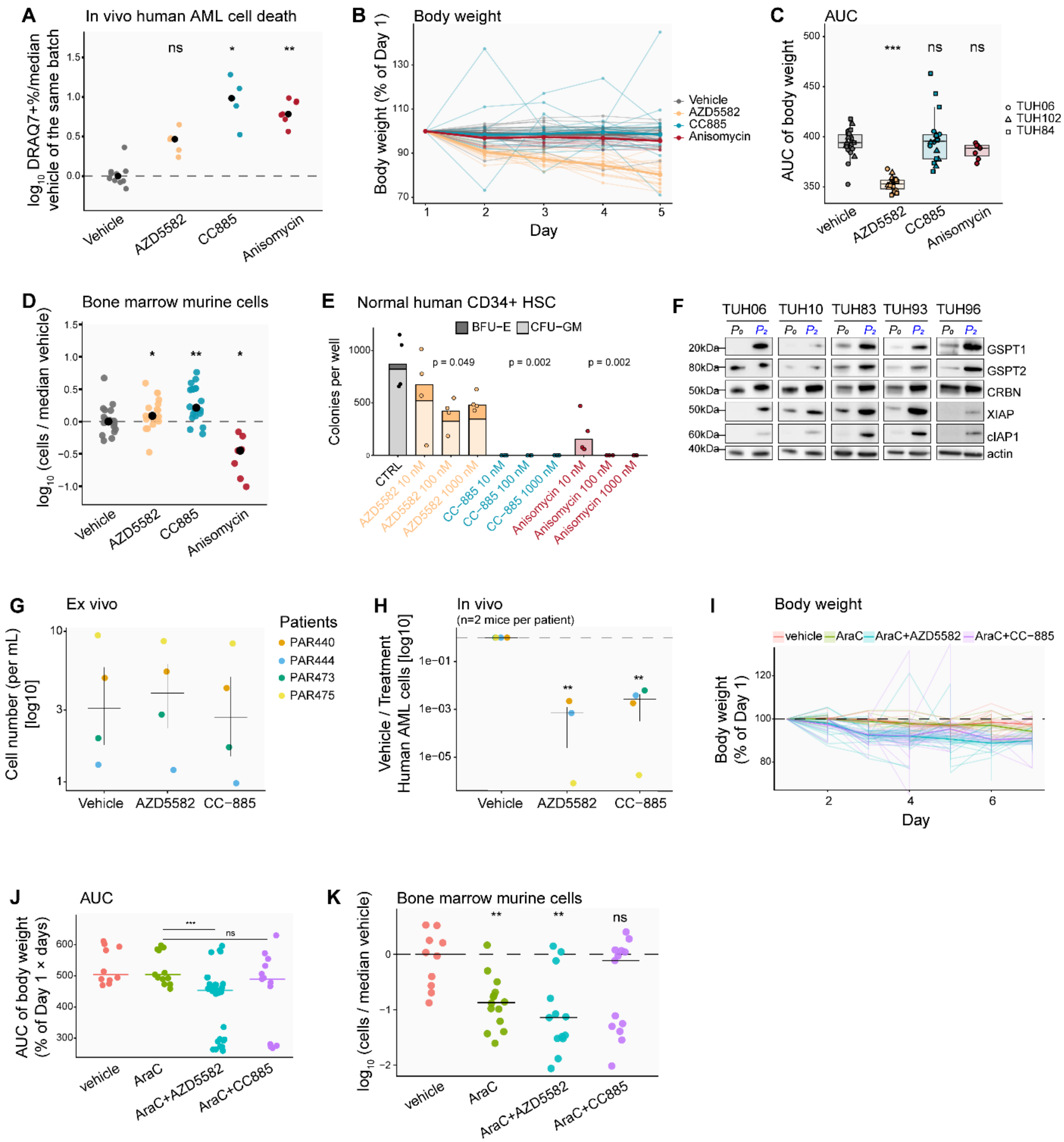

Figure S6. Targeting evolution-enriched dependencies suppresses leukemic propagation in vivo. Related to Figure 6. A. In vivo human AML cell death following treatment with vehicle, AZD5582 (2 mg/kg/day, intraperitoneal), CC-885 (10 mg/kg/day, intraperitoneal), or anisomycin (10mg/kg/day, intraperitoneal) in TUH06 PDXs. Cell death was measured by DRAQ7 staining and is shown after within-batch normalization. **B.** Body weight trajectories during 7-day treatment with vehicle, AZD5582, CC-885, or anisomycin in PDX-bearing mice. Body weight is expressed as percentage of day 1. **C.** Area under the curve (AUC) calculated for each mouse from longitudinal body weight measurements using the trapezoidal rule. Values represent integrated body weight over time (% of day 1 × days). **D.** Murine bone marrow cellularity after single-agent treatment. AZD5582 and CC-885

were evaluated across three PDX models, whereas anisomycin was evaluated in TUH06 only. Normalization was performed within each PDX model. **E.** Clonogenic capacity of normal human $CD34^{+}$ hematopoietic stem and progenitor cells following exposure to AZD5582, CC-885, or anisomycin. Colonies were quantified at day 10 in methylcellulose cultures and are shown as total colonies per well. Bar plots represent median colony output with subdivision into BFU-E and CFU-GM compartments (stacked), while overlaid points indicate total colony counts per replicate. Statistical significance was assessed using Kruskal–Wallis tests within each compound (including vehicle). **F.** Baseline protein expression in P_0 vs. P_2 samples from five different patients, probed for GSPT1, GSPT2, CRBN, XIAP, cIAP1, and β-actin (loading control). **G-H.** Ex vivo and in vivo assessment of primary AML samples treated with vehicle, AZD5582, or CC-885. **G.** Viable cell number after 48-hour ex vivo exposure. **H.** Ratio of human AML cells recovered in vivo (Vehicle/Treatment; log10) following transplantation (n=2 mice per patient; patients indicated). **I.** Body weight trajectories under combination therapy: vehicle, Ara-C (30 mg/kg/day), Ara-C + AZD5582 (2 mg/kg/day), Ara-C + CC-885 (10 mg/kg/day); intraperitoneal, 7 days. Body weight is expressed as percentage of day 1. **J.** AUC calculated for each mouse from the longitudinal body weight measurements using the trapezoidal rule. **K.** Murine bone marrow cellularity under combination treatment, expressed as log10(cells / median vehicle). Each dot represents one mouse. Black dots indicate group medians where shown. ns: not significant; *$p<0.05$; **$p<0.01$; ***$p<0.001$.

Patient	G	Age	W	B	FAB	Karyotype	NGS	EL	T	E
PAR440	F	78	65		AML1				N	Y
PAR444	F	74	34		s-AML	del(5q)	*PTPN11*	A	N	Y
PAR473	M		17	76	s-AML	N	*IDH2*, *ASXL1*, *NRAS*	A	N	Y
PAR475	F	76	200	100	AML1	N	*FLT3*, *NPM1*	I	N	Y

Table S17. Clinical and molecular characteristics of patients with AML. Related to Figure 6. Patients are anonymized and identified by a unique code in the Patient column. G: Gender. M, male; F, female. Age: Age in years at time of sample collection. W: white blood cell count ($\times 10^9$/L). B: Percentage of blast cells in the sample. FAB: French-American-British (FAB) classification subtypes. s-AML: secondary AML. Karyotype. N: normal karyotype. del(5q): del(5)(q13q33). NGS: next-generation sequencing analysis of a panel of genes associated with myeloid neoplasms. ELN 2022 risk classification according to the European LeukemiaNet: F = favorable, I = intermediate, A = adverse. T: serial transplantation assays. Ex: ex vivo followed by xenotransplantation assays. Y: yes. N: no.
